# Supplementary material for: Nutrient deprivation alters the rate of COPII subunit recruitment at ER subdomains to tune secretory protein transport
Source: Nat Commun. 2023 Dec 8;14:8140. doi: 10.1038/s41467-023-44002-7 (PMC10709328; doi:10.1038/s41467-023-44002-7)
Supplement: Supplementary file 1 — Supplementary Information [file 41467_2023_44002_MOESM1_ESM.pdf]

Supplementary Information

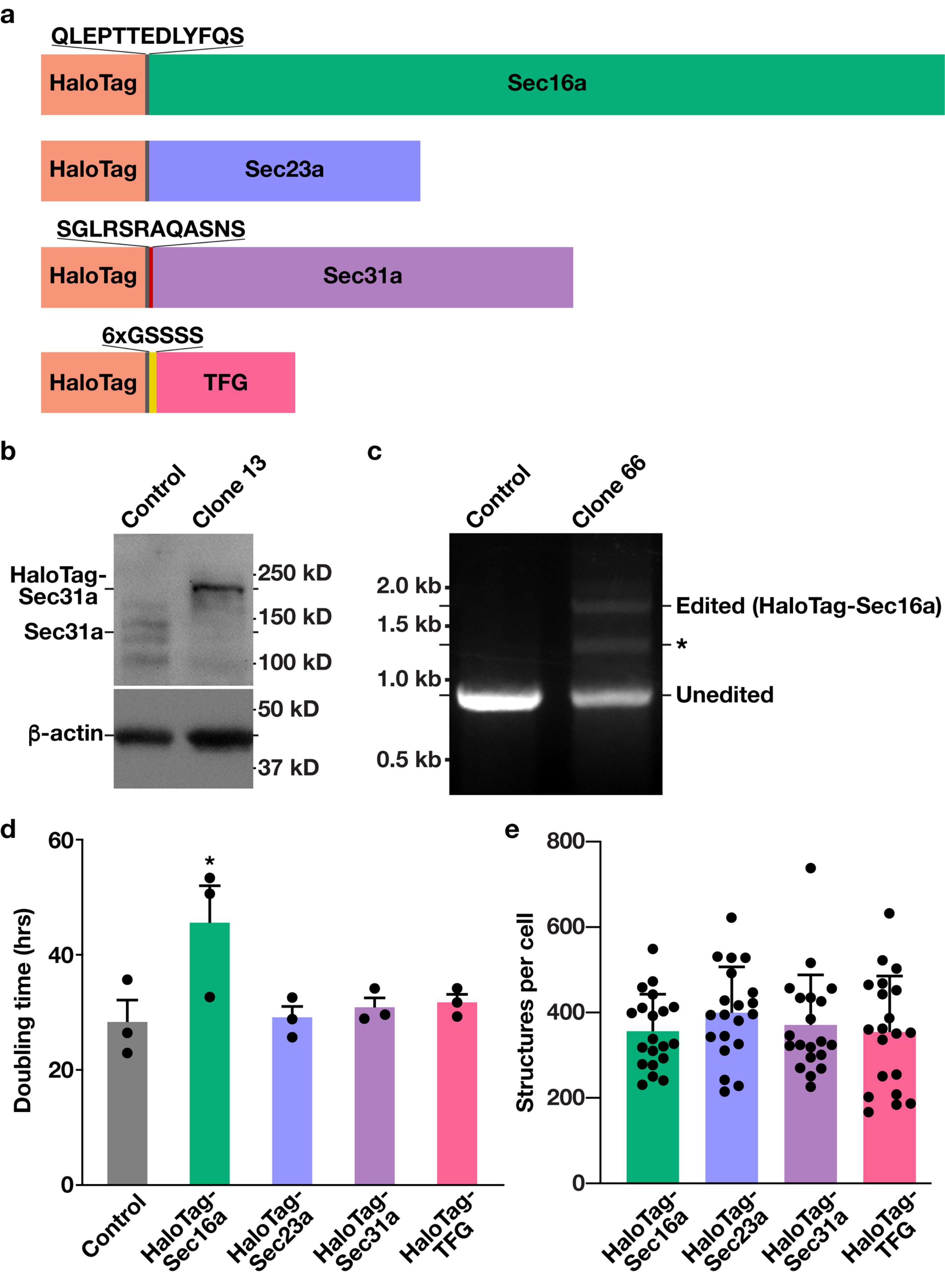

a
QLEPTTEDLYFQS
HaloTag
Sec16a
HaloTag
Sec23a
SGLRSRAQASNS
HaloTag
Sec31a
6xGSSSS
HaloTag
TFG
b
Control
Clone 13
HaloTag-Sec31a
Sec31a
β-actin
250 kD
150 kD
100 kD
50 kD
37 kD
c
Control
Clone 66
2.0 kb
1.5 kb
1.0 kb
0.5 kb
Edited (HaloTag-Sec16a)
*
Unedited
d
Doubling time (hrs)
60
40
20
0
*
Control
HaloTag-Sec16a
HaloTag-Sec23a
HaloTag-Sec31a
HaloTag-TFG
e
Structures per cell
800
600
400
200
0
HaloTag-Sec16a
HaloTag-Sec23a
HaloTag-Sec31a
HaloTag-TFG

Figure S1. Appending HaloTag onto endogenous COPII subunits fails to impact their function. (A) Cartoons depicting the strategy used to create HaloTag (orange) fusions with native Sec16a (green), Sec23a (light blue) (62), Sec31a (purple), and TFG (salmon). (B) Representative immunoblots (n=3) of extracts generated from control and clonal CRISPR/Cas9 edited cell lines using antibodies directed against Sec31a (top) and β-actin (bottom). (C) PCR was used to analyze genomic DNA from control cells and clonal cells expressing a HaloTag fusion to Sec16a (n=3). An asterisk highlights a nonspecific band, based on sequence analysis. (D) The proliferation rates of control cells (grey) and genome edited cells expressing HaloTag fusion proteins (HaloTag-Sec16a, green; HaloTag-Sec23a, light blue; HaloTag-Sec31a, purple; HaloTag-TFG, salmon) were determined using widefield imaging of Calcein AM, which marks living cells. Error bars represent the mean +/- SEM (n=3 biological replicates each). *, p = 0.0315 as calculated using a one-way ANOVA and Tukey post hoc test. (E) Based on confocal microscopy, the total number of structures decorated by each HaloTag fusion protein (HaloTag-Sec16a, green; HaloTag-Sec23a, light blue; HaloTag-Sec31a, purple; HaloTag-TFG, salmon) following labeling with JFX650-HaloTag ligand was calculated throughout the volume of cells (n=20 cells each; 3 biological replicates each). No statistically significant differences were found, as calculated using a one-way ANOVA and Tukey post hoc test.

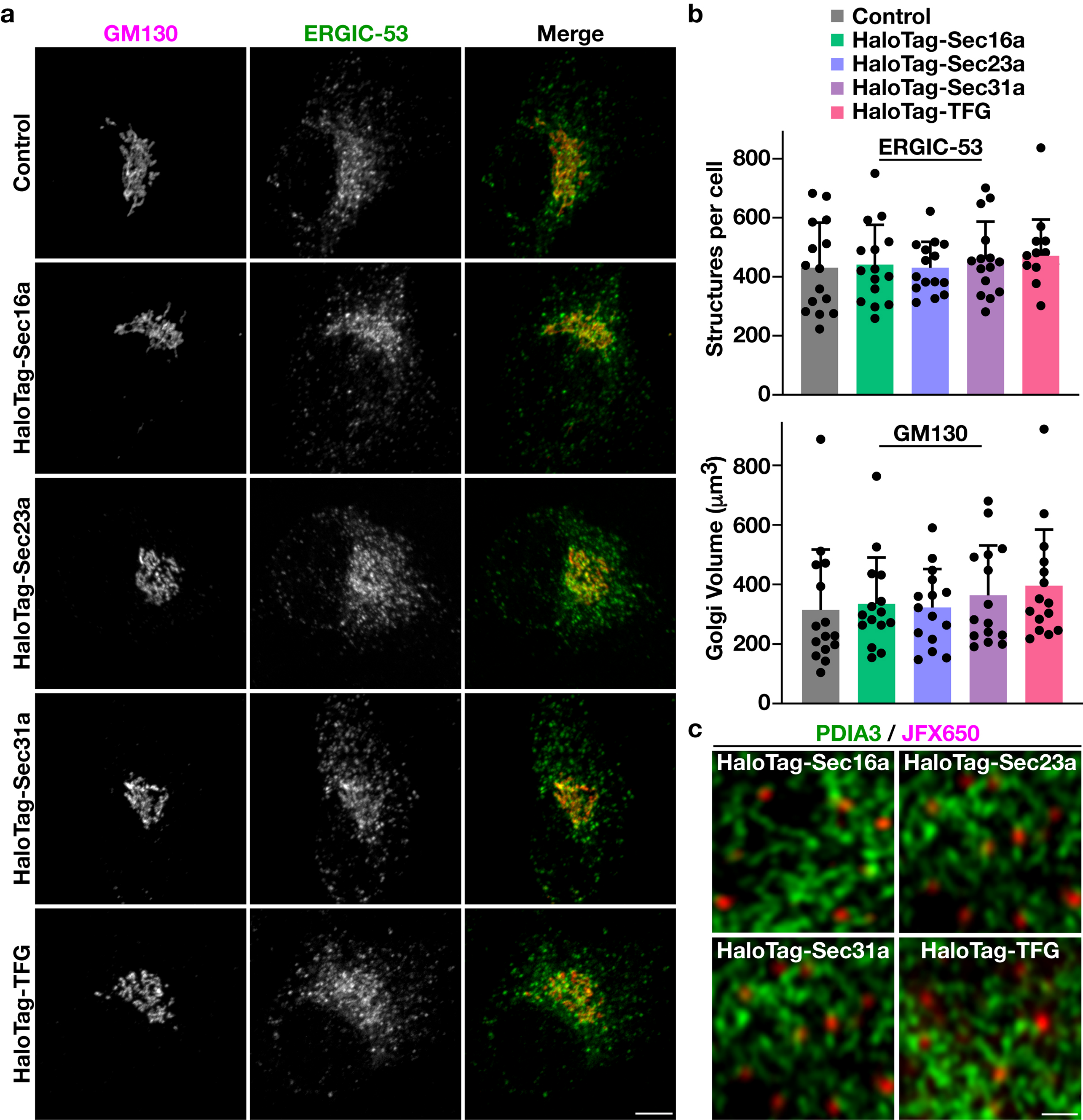
a
GM130
ERGIC-53
Merge
Control
HaloTag-Sec16a
HaloTag-Sec23a
HaloTag-Sec31a
HaloTag-TFG
b
Control
HaloTag-Sec16a
HaloTag-Sec23a
HaloTag-Sec31a
HaloTag-TFG
ERGIC-53
Structures per cell
800
600
400
200
0
GM130
Golgi Volume (μm3)
800
600
400
200
0
c
PDIA3 / JFX650
HaloTag-Sec16a
HaloTag-Sec23a
HaloTag-Sec31a
HaloTag-TFG

Figure S2. Genome edited cell lines exhibit normal organization of the early secretory pathway. (A) Representative confocal images of cell lines natively expressing HaloTag fusion proteins co-stained with antibodies directed against endogenous GM130 (magenta) and ERGIC-53 (green). Bar, 10 μm. (B) Based on confocal microscopy, the total number of structures decorated by ERGIC-53 (top) and the total cellular volume labeled by GM130 (bottom) was calculated within control cells (grey) and cells expressing HaloTag fusions proteins (HaloTag-Sec16a, green; HaloTag-Sec23a, light blue; HaloTag-Sec31a, purple; HaloTag-TFG, salmon) (n=15 cells each; 3 biological replicates each). No statistically significant differences were found, as calculated using a one-way ANOVA and Tukey post hoc test. (C) Representative super resolution images of fixed cells expressing HaloTag fusion proteins labeled with the JFX650-HaloTag ligand (magenta) and co-stained using antibodies directed against PDIA3 (green). Bar, 1 μm.

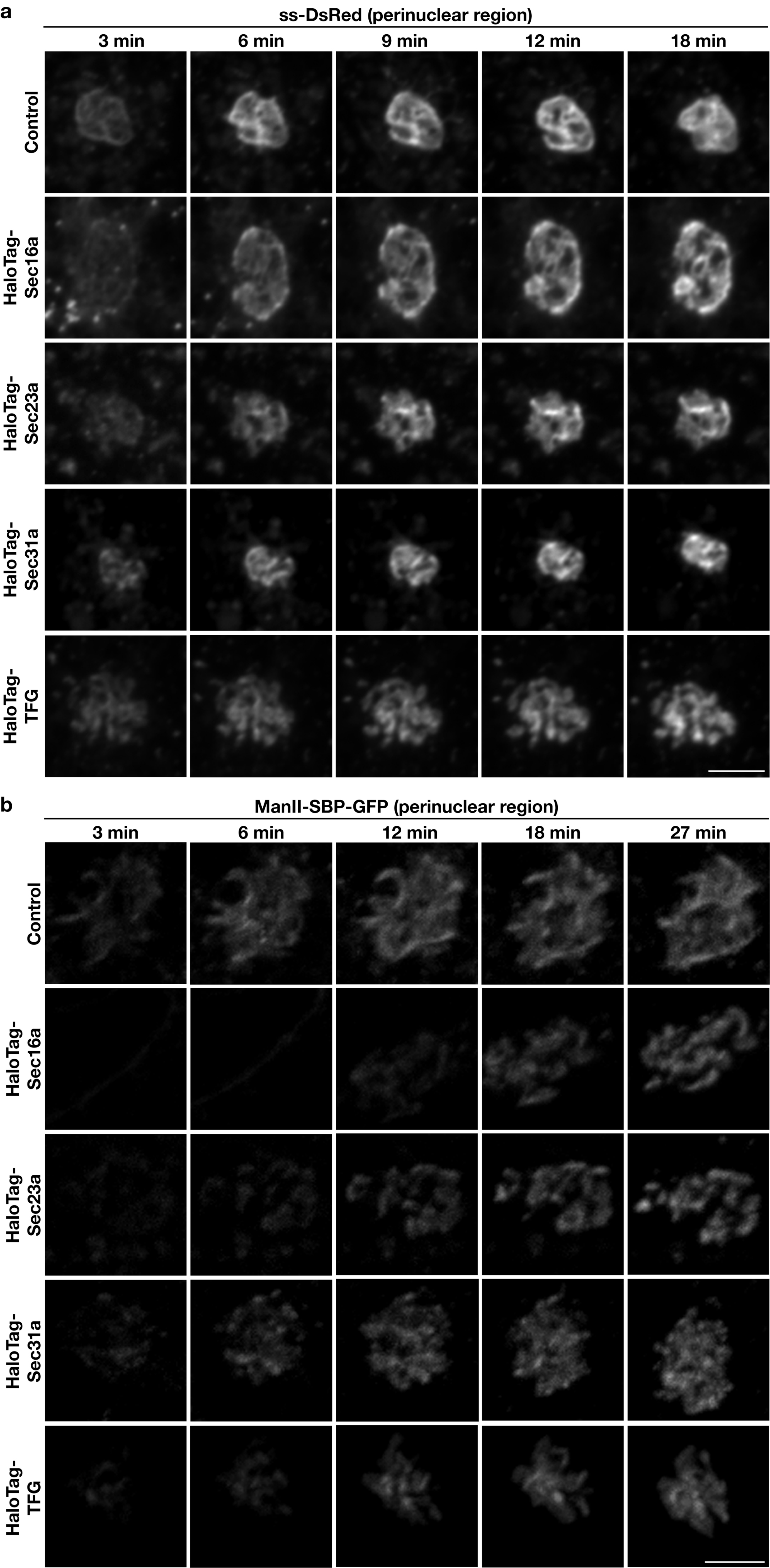
a
ss-DsRed (perinuclear region)
3 min
6 min
9 min
12 min
18 min
Control
HaloTag-Sec16a
HaloTag-Sec23a
HaloTag-Sec31a
HaloTag-TFG
b
ManII-SBP-GFP (perinuclear region)
3 min
6 min
12 min
18 min
27 min
Control
HaloTag-Sec16a
HaloTag-Sec23a
HaloTag-Sec31a
HaloTag-TFG

Figure S3. Genome edited cell lines exhibit normal trafficking of secretory cargoes. (A and B) Representative confocal images of control cells and cell lines natively expressing HaloTag fusion proteins co-expressing ss-DsRed (A) or ManII-SBP-GFP (B) at various timepoints following their release from the ER (n=15 cells each; 3 biological replicates each). Zoomed images of the perinuclear region are shown. Bar, 5 μm.

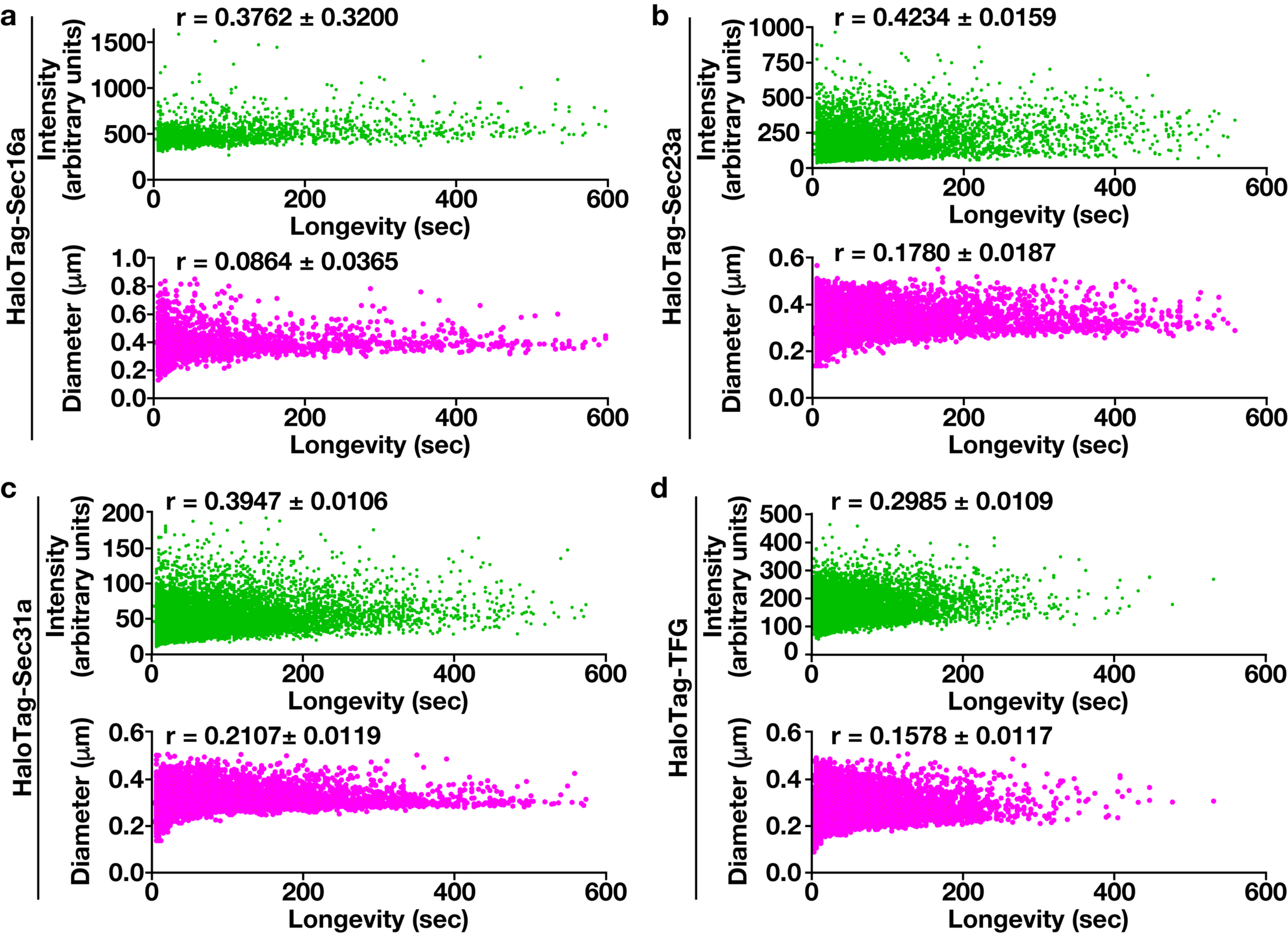
a
HaloTag-Sec16a
r = 0.3762 ± 0.3200
Intensity (arbitrary units)
Longevity (sec)
r = 0.0864 ± 0.0365
Diameter (μm)
Longevity (sec)
b
HaloTag-Sec23a
r = 0.4234 ± 0.0159
Intensity (arbitrary units)
Longevity (sec)
r = 0.1780 ± 0.0187
Diameter (μm)
Longevity (sec)
c
HaloTag-Sec31a
r = 0.3947 ± 0.0106
Intensity (arbitrary units)
Longevity (sec)
r = 0.2107± 0.0119
Diameter (μm)
Longevity (sec)
d
HaloTag-TFG
r = 0.2985 ± 0.0109
Intensity (arbitrary units)
Longevity (sec)
r = 0.1578 ± 0.0117
Diameter (μm)
Longevity (sec)

Figure S4. Long-lived structures harboring HaloTag fusion proteins exhibit increased intensity and size relative to short-lived sites. (A-D) Pearson's correlation analysis was used to calculate the covariance (represented by correlation coefficient, r) between site longevity and either intensity (top; green) or diameter (bottom; magenta) for structures harboring HaloTag fusion proteins (n=10 cells each; 3 biological replicates; more than 3000 particles each).

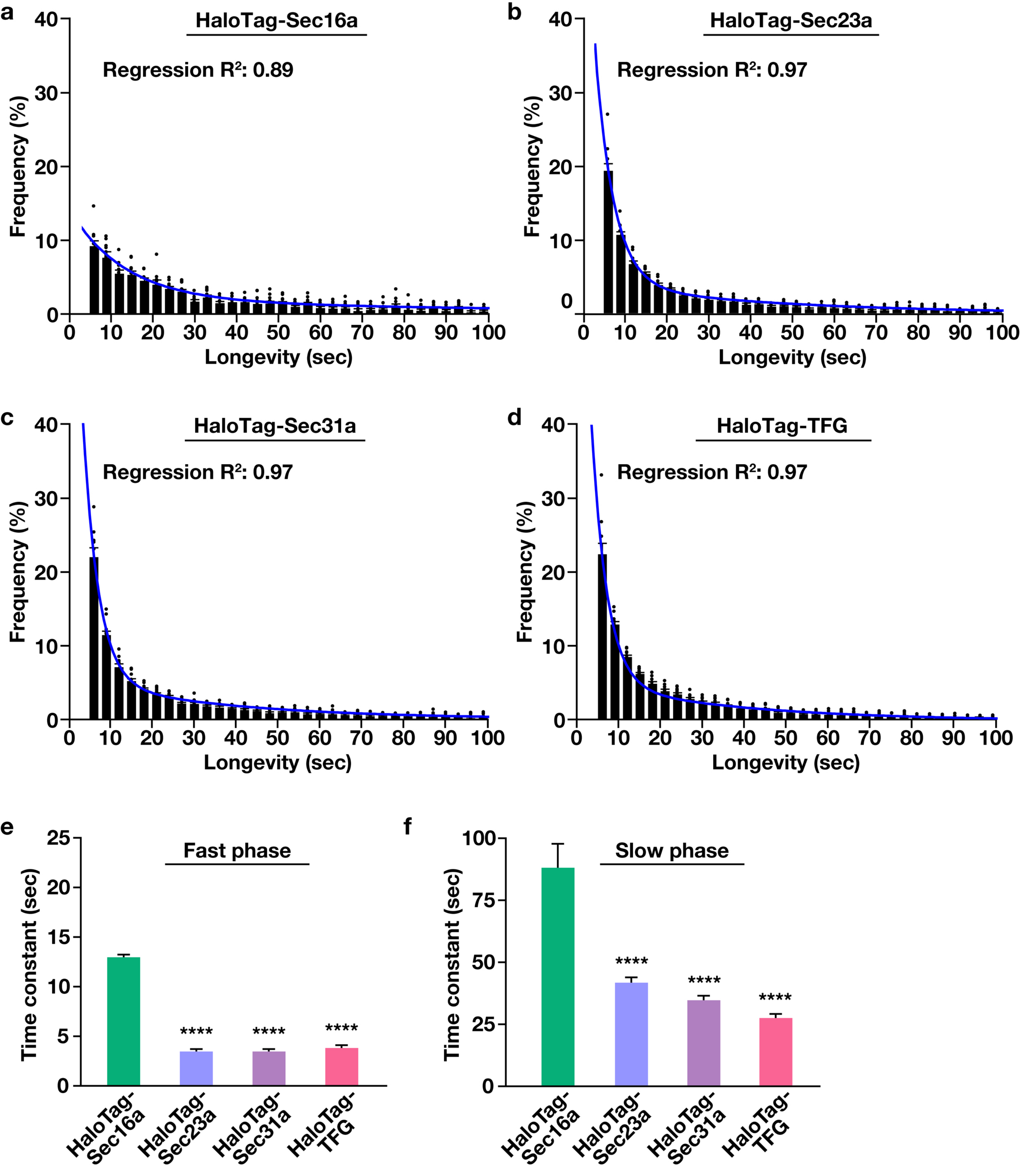

a
HaloTag-Sec16a
Regression R²: 0.89
Frequency (%)
Longevity (sec)
b
HaloTag-Sec23a
Regression R²: 0.97
Frequency (%)
Longevity (sec)
c
HaloTag-Sec31a
Regression R²: 0.97
Frequency (%)
Longevity (sec)
d
HaloTag-TFG
Regression R²: 0.97
Frequency (%)
Longevity (sec)
e
Fast phase
Time constant (sec)
****
HaloTag-Sec16a
HaloTag-Sec23a
HaloTag-Sec31a
HaloTag-TFG
f
Slow phase
Time constant (sec)
****
HaloTag-Sec16a
HaloTag-Sec23a
HaloTag-Sec31a
HaloTag-TFG

Figure S5. At least two types of sites harboring each HaloTag fusion protein are present in cells. (A-D) Based on lattice light-sheet imaging, histograms were generated to describe how frequently sites of differing longevity were found. Blue curves demonstrate fitting to a two-phase exponential decay equation (R^2 values shown). Error bars represent mean +/- SEM (n=10 cells each; 3 biological replicates; more than 3000 tracked particles each). (E and F) Based on two-phase exponential decay regressions for each HaloTag fusion protein (HaloTag-Sec16a, green; HaloTag-Sec23a, light blue; HaloTag-Sec31a, purple; HaloTag-TFG, salmon), time constants for distinct fast (E) and slow (F) phases were calculated. Error bars represent mean +/- SEM (n=10 cells each; more than 3000 structures each; 3 biological replicates). ****, p = 0.0001, calculated using a one-way ANOVA and Tukey post hoc test.

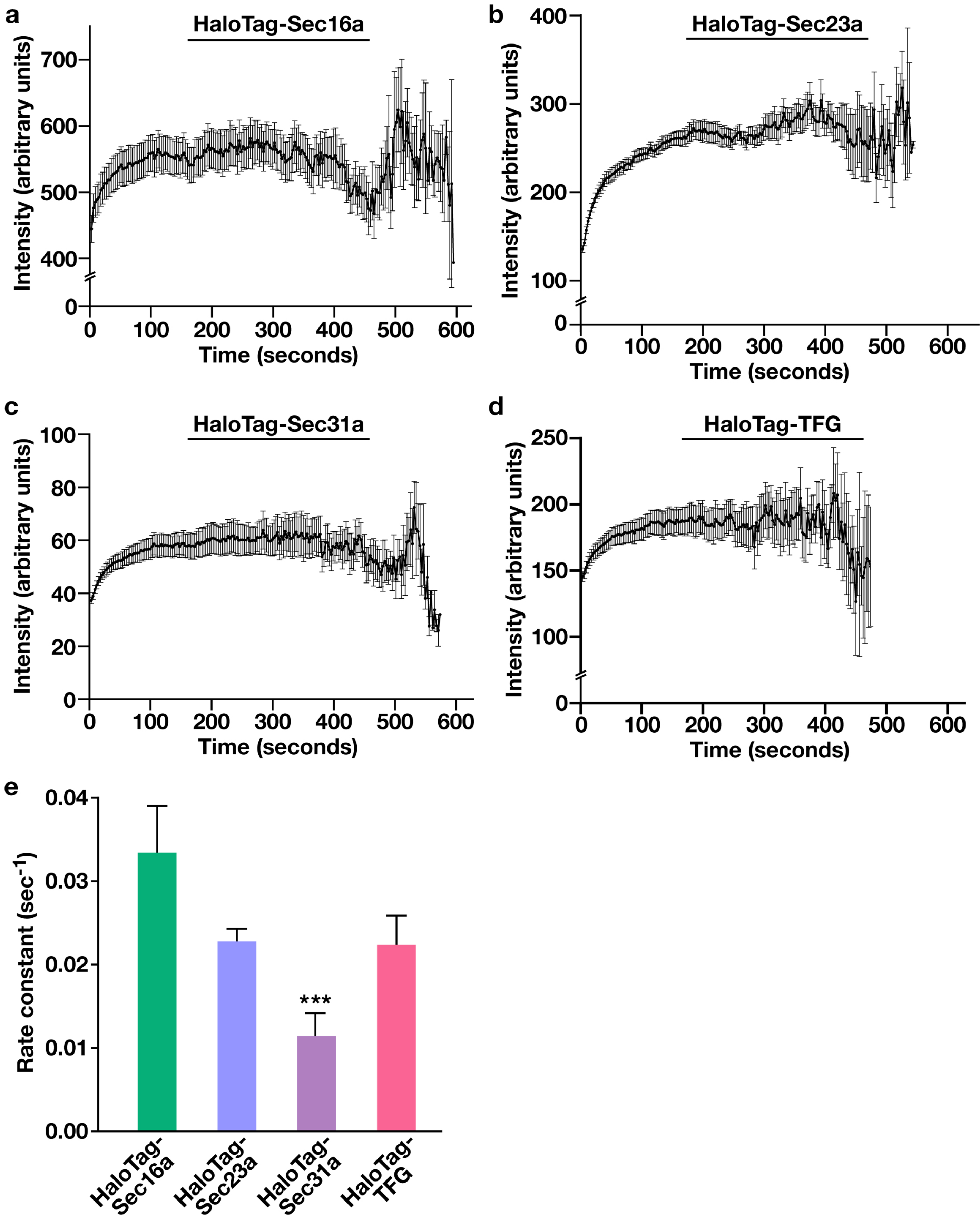
a
HaloTag-Sec16a
Intensity (arbitrary units)
Time (seconds)
b
HaloTag-Sec23a
Intensity (arbitrary units)
Time (seconds)
c
HaloTag-Sec31a
Intensity (arbitrary units)
Time (seconds)
d
HaloTag-TFG
Intensity (arbitrary units)
Time (seconds)
e
Rate constant (sec-1)
***
HaloTag-Sec16a
HaloTag-Sec23a
HaloTag-Sec31a
HaloTag-TFG

Figure S6. Dynamics of HaloTag fusion proteins at ER subdomains. (A-D) Fluorescence intensity of HaloTag-Sec16a (A), HaloTag-Sec23a (B), HaloTag-Sec31a (C), and HaloTag-TFG (D) positive structures tracked over time using light-sheet imaging following labeling with JFX650-HaloTag ligand. Error bars represent mean +/- SEM (n=10 cells each; more than 3000 structures each; 3 biological replicates). (E) Rate constants were calculated based on assembly curves for each HaloTag fusion protein (HaloTag-Sec16a, green; HaloTag-Sec23a, light blue; HaloTag-Sec31a, purple; HaloTag-TFG, salmon, fitted to an exponential plateau equation). Error bars represent mean +/- SEM (n=10 cells each; more than 3000 structures each; 3 biological replicates). ***, p = 0.0009, calculated using a one-way ANOVA and Tukey post hoc test.

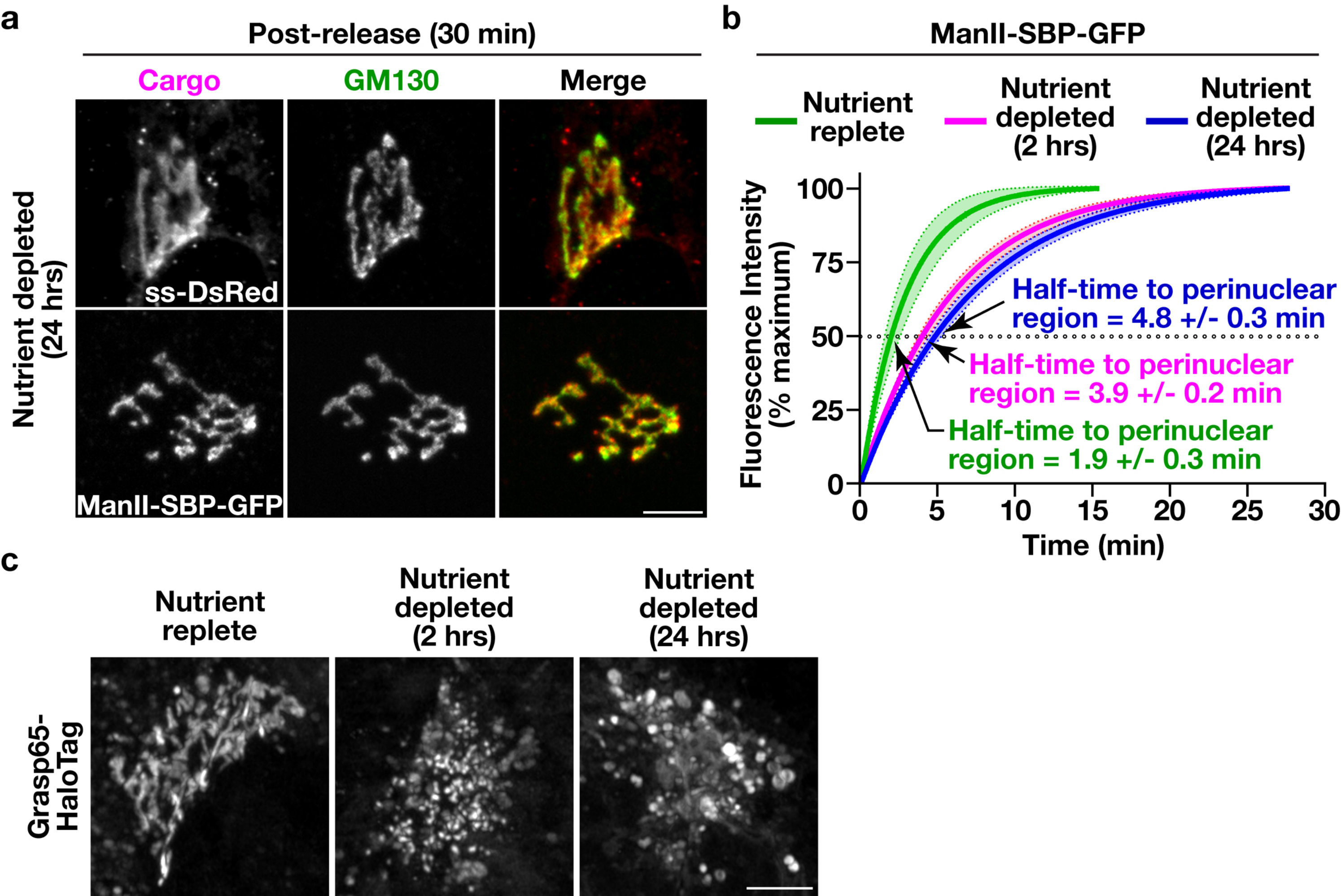

a
Post-release (30 min)
Cargo
GM130
Merge
Nutrient depleted (24 hrs)
ss-DsRed
ManII-SBP-GFP
b
ManII-SBP-GFP
Nutrient replete
Nutrient depleted (2 hrs)
Nutrient depleted (24 hrs)
Fluorescence Intensity (% maximum)
Time (min)
Half-time to perinuclear region = 4.8 +/- 0.3 min
Half-time to perinuclear region = 3.9 +/- 0.2 min
Half-time to perinuclear region = 1.9 +/- 0.3 min
c
Nutrient replete
Nutrient depleted (2 hrs)
Nutrient depleted (24 hrs)
Grasp65-HaloTag

Figure S7. Acute and long-term nutrient deprivation similarly influence the rate of ManII-SBP-GFP trafficking. (A) Confocal imaging of control cells expressing ss-DsRed or ManII-SBP-GFP (magenta) was used to monitor their accumulation at Golgi membranes harboring GM130 (green) following their release from the ER under prolonged nutrient deprivation conditions. Bar, 5 μm. (B) Based on fluorescence intensity, the percentage of ManII-SBP-GFP present within the perinuclear region relative to its maximal accumulation there was quantified over time and fitted to an exponential with the dotted line indicating half-maximal intensity. Error, as displayed by lightly colored bands, represent mean +/- SEM (n=30 cells each; 3 biological replicates each). Half-times to perinuclear accumulation of ss-DsRed were calculated (see Source Data file) based on the plots under various nutrient availability conditions (nutrient replete, green; 2 hour nutrient depletion, magenta; 24 hour nutrient depletion, blue). (C) Representative super resolution images of clonal CRISPR/Cas9-edited cells expressing GRASP65-HaloTag in the presence and absence of nutrients following labeling with JFX650-HaloTag ligand. Bar, 5 μm.

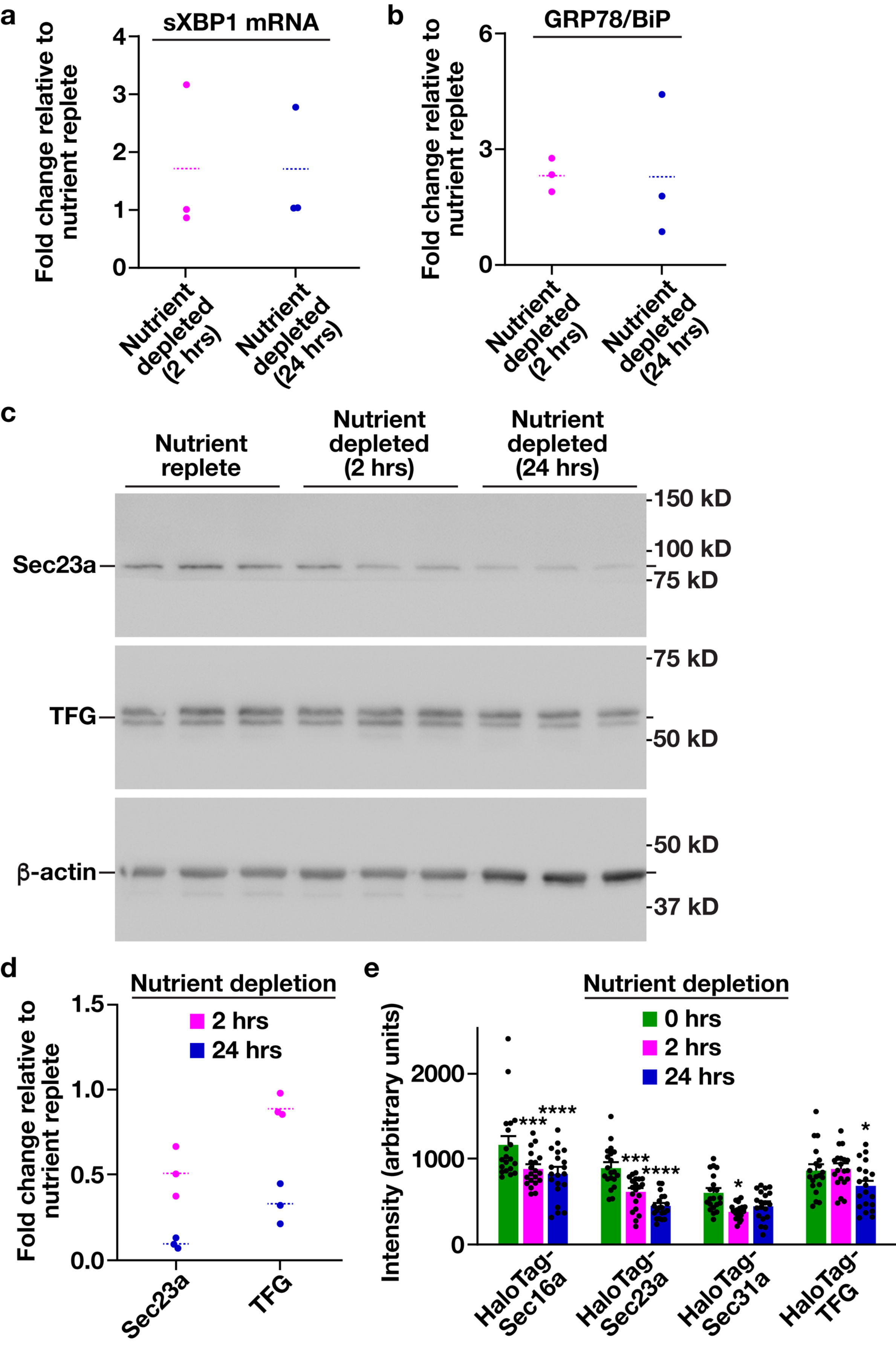

a
sXBP1 mRNA
Fold change relative to nutrient replete
4
3
2
1
0
Nutrient depleted (2 hrs)
Nutrient depleted (24 hrs)
b
GRP78/BiP
Fold change relative to nutrient replete
6
3
0
Nutrient depleted (2 hrs)
Nutrient depleted (24 hrs)
c
Nutrient replete
Nutrient depleted (2 hrs)
Nutrient depleted (24 hrs)
Sec23a
TFG
β-actin
150 kD
100 kD
75 kD
75 kD
50 kD
50 kD
37 kD
d
Nutrient depletion
2 hrs
24 hrs
Fold change relative to nutrient replete
1.5
1.0
0.5
0.0
Sec23a
TFG
e
Nutrient depletion
0 hrs
2 hrs
24 hrs
Intensity (arbitrary units)
2000
1000
0
***
****
***
****
*
*
HaloTag-Sec16a
HaloTag-Sec23a
HaloTag-Sec31a
HaloTag-TFG

Figure S8. Nutrient deprivation reduces expression of COPII subunits. (A) Levels of sXBP1 mRNA in nutrient deprived cells were determined relative to control cells (maintained in the presence of nutrients) using quantitative PCR (2 hour nutrient depletion, magenta; 24 hour nutrient depletion, blue; n=3 biological replicates). No statistically significant differences were found, as calculated using an unpaired *t* test. (B) Levels of GRP78 in nutrient deprived cells were determined relative to control cells (maintained in the presence of nutrients) using quantitative immunoblotting (2 hour nutrient depletion, magenta; 24 hour nutrient depletion, blue; n=3 biological replicates). No statistically significant differences were found, as calculated using an unpaired *t* test. (C) Representative immunoblots (n=3 each) of extracts generated from control cells in the presence and absence of nutrients using antibodies directed against Sec23a (top), TFG (middle) and β-actin (bottom). (D) Quantification of immunoblots shown in panel C (2 hour nutrient depletion, magenta; 24 hour nutrient depletion, blue; 3 biological replicates). (E) Based on quantitative cellular fluorescence measurements, the intensity of each HaloTag fusion protein was compared in the presence and absence of nutrients (nutrient replete, green; 2 hour nutrient depletion, magenta; 24 hour nutrient depletion, blue). Error bars represent mean +/- SEM (n=20 cells each; 3 biological replicates each). ****p = 0.001, ***p = 0.006 (HaloTag-Sec16a nutrient replete as compared to 2 hour nutrient deprivation) or 0.005 (HaloTag-Sec23a nutrient replete as compared to 2 hour nutrient deprivation), and *p = 0.0221 (HaloTag-Sec31a nutrient replete as compared to 2 hour nutrient deprivation) or 0.0267 (HaloTag-TFG nutrient replete as compared to 24 hour nutrient deprivated), each calculated using a one-way ANOVA and Tukey post hoc test.

.

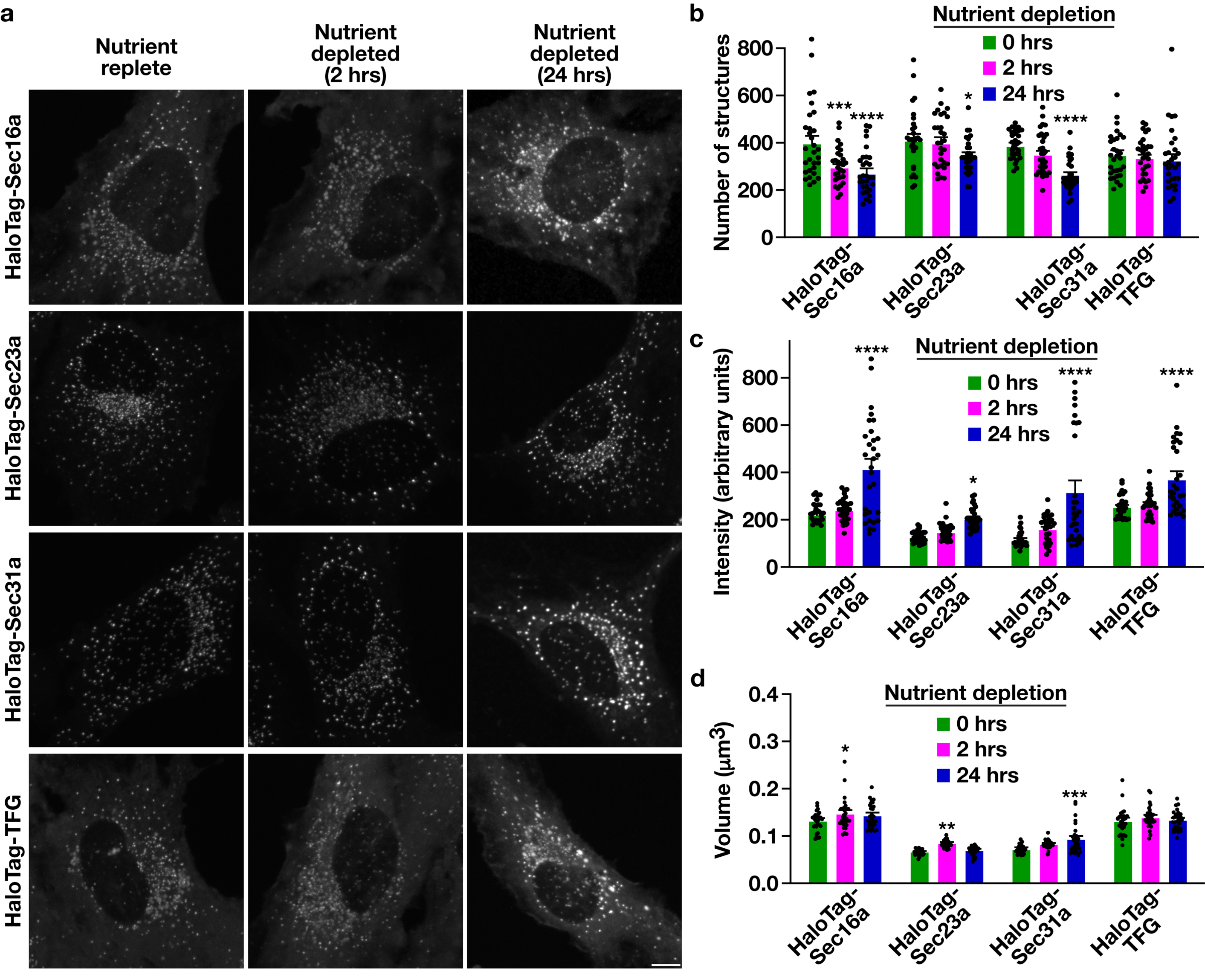
a
Nutrient replete
Nutrient depleted (2 hrs)
Nutrient depleted (24 hrs)
HaloTag-Sec16a
HaloTag-Sec23a
HaloTag-Sec31a
HaloTag-TFG
b
Nutrient depletion
0 hrs
2 hrs
24 hrs
Number of structures
800
600
400
200
0
***
****
*
****
HaloTag-Sec16a
HaloTag-Sec23a
HaloTag-Sec31a
HaloTag-TFG
c
Nutrient depletion
0 hrs
2 hrs
24 hrs
Intensity (arbitrary units)
800
600
400
200
0
****
*
****
****
HaloTag-Sec16a
HaloTag-Sec23a
HaloTag-Sec31a
HaloTag-TFG
d
Nutrient depletion
0 hrs
2 hrs
24 hrs
Volume (μm3)
0.4
0.3
0.2
0.1
0.0
*
**
***
HaloTag-Sec16a
HaloTag-Sec23a
HaloTag-Sec31a
HaloTag-TFG

Figure S9. Long-term nutrient deprivation reduces the number of COPII-positive structures in cells, but elevates their intensities. (A) Representative confocal images of cell lines natively expressing HaloTag fusion proteins after labeling with JFX650-HaloTag ligand in the presence and absence of nutrients are shown. Bar, 5 μm. (B-D) Based on confocal microscopy, the total number of structures decorated by each HaloTag fusion protein (B), their intensities (C) and their volumes (D) were calculated in the presence and absence of nutrients (nutrient replete, green; 2 hour nutrient depletion, magenta; 24 hour nutrient depletion, blue). Error bars represent mean +/- SEM (n=30 cells each; 3 biological replicates each). ****p = 0.0001 and *p = 0.0163 (panel B), ****p = 0.0001 and *p = 0.0116 (panel C), and ***p = 0.0003, **p = 0.0017, and *p = 0.0184 (panel D), each calculated using a one-way ANOVA and Tukey post hoc test. Additional p values for other comparisons are provided in the Source Data file.

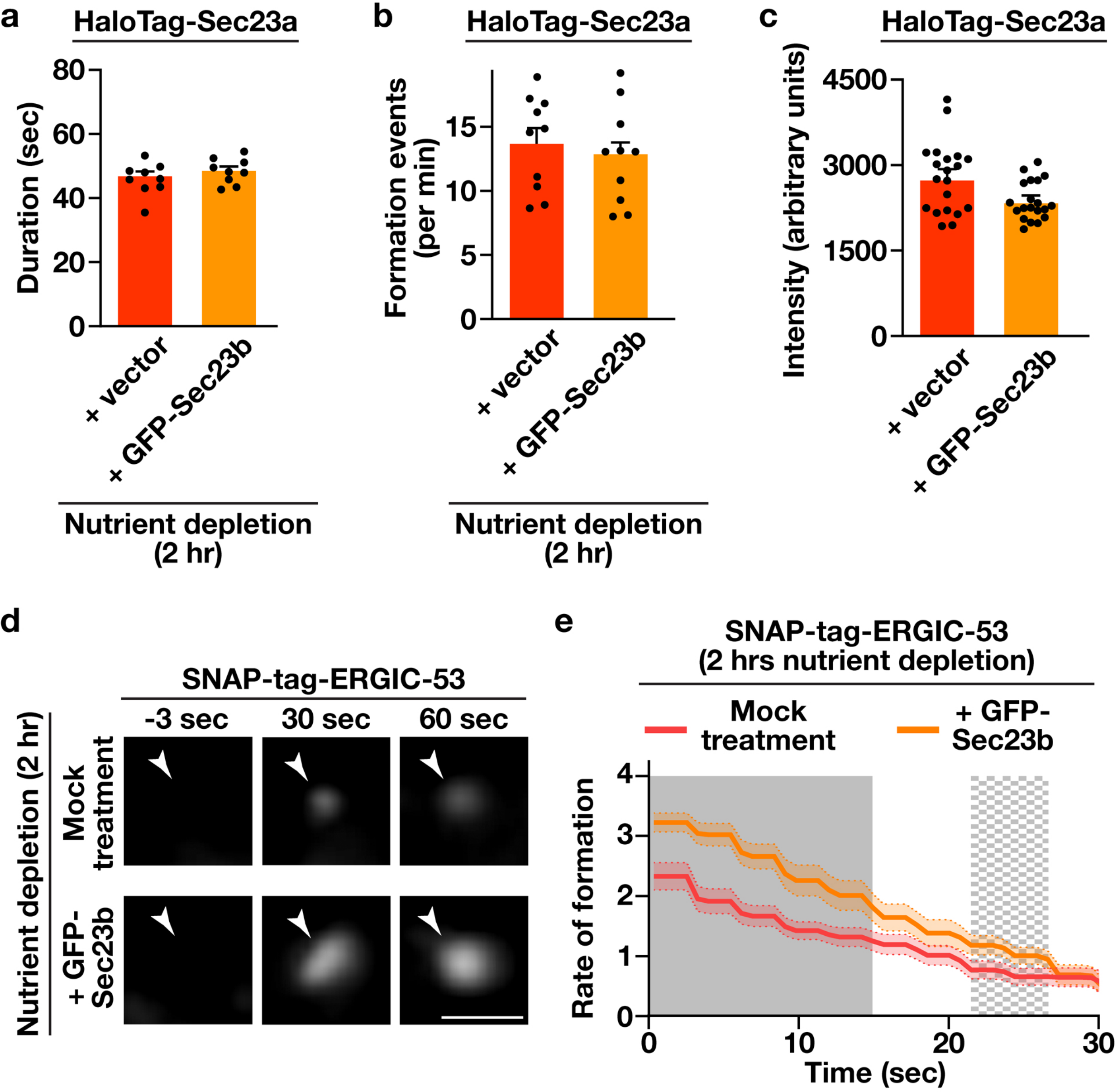
a
HaloTag-Sec23a
Duration (sec)
80
60
40
20
0
+ vector
+ GFP-Sec23b
Nutrient depletion
(2 hr)
b
HaloTag-Sec23a
Formation events
(per min)
15
10
5
0
+ vector
+ GFP-Sec23b
Nutrient depletion
(2 hr)
c
HaloTag-Sec23a
Intensity (arbitrary units)
4500
3000
1500
0
+ vector
+ GFP-Sec23b
d
SNAP-tag-ERGIC-53
-3 sec
30 sec
60 sec
Nutrient depletion (2 hr)
Mock
treatment
+ GFP-
Sec23b
e
SNAP-tag-ERGIC-53
(2 hrs nutrient depletion)
Mock
treatment
+ GFP-
Sec23b
Rate of formation
4
3
2
1
0
0
10
20
30
Time (sec)

Figure S10. Overexpression of GFP-Sec23b accelerates cargo trafficking from the ER in cells depleted acutely of nutrients. (A) Quantification of the average duration of HaloTag-Sec23a at ER subdomains in the presence (yellow) or absence (orange) of overexpressed GFP-Sec23b following acute nutrient deprivation. Error bars represent mean +/- SEM (n=20 cells each; 3 biological replicates). No statistically significant difference was found, as calculated using an unpaired *t* test. (B) Quantification of the number of HaloTag-Sec23a positive structures that form each minute in the presence (yellow) or absence (orange) of overexpressed GFP-Sec23b following acute nutrient deprivation. Error bars represent mean +/- SEM (n=20 cells each; 3 biological replicates). No statistically significant differences were found, as calculated using an unpaired *t* test. (C) The total cellular intensity of HaloTag-Sec23a following labeling with JFX650-HaloTag ligand was determined in the presence (yellow) or absence (orange) of overexpressed GFP-Sec23b. Error bars represent mean +/- SEM (n=20 cells each; 3 biological replicates). No statistically significant difference was found, as calculated using an unpaired *t* test. (D) Following nutrient depletion (2 hrs), HILO imaging was used to monitor the de novo formation of sites harboring SNAP-tag-ERGIC-53 (highlighted by arrowheads) in the presence or absence of GFP-Sec23b. Bar, 1 μm. (E) The rates at which sites harboring SNAP-tag-ERGIC-53 formed in the presence and absence of exogenously expressed GFP-Sec23b were derived from assembly curves generated from data described in panel D. Error, as displayed by lightly colored bands, represents mean +/- SEM (n=15 cells each; more than 3000 structures each; 3 biological replicates). Solid gray ($p < 0.05$) and checkered gray ($p < 0.1$) regions represent significant differences, calculated using multiple, unpaired *t* tests at each time point. Specific p values are provided in the Source Data file.
