# Supplementary material for: Nutrient deprivation alters the rate of COPII subunit recruitment at ER subdomains to tune secretory protein transport
Source: Nat Commun. 2023 Dec 8;14:8140. doi: 10.1038/s41467-023-44002-7 (PMC10709328; doi:10.1038/s41467-023-44002-7)
Supplement: Supplementary file 3 — Description of Additional Supplementary Files [file 41467_2023_44002_MOESM3_ESM.pdf]

Description of Additional Supplementary Files

Supplementary Movies 1-4. Dynamics of HaloTag-Sec16a (Supplementary Movie 1), HaloTag-Sec23a (Supplementary Movie 2), HaloTag-Sec31a (Supplementary Movie 3), and HaloTag-TFG (Supplementary Movie 4) as determined using lattice light-sheet imaging (n=10 cells each; 3 biological replicates each). Bar, 10 µm. Playback rate, 180x.

Supplementary Movies 5-7. Accumulation of ss-DsRed in the perinuclear region of control cells following its release in the presence (Supplementary Movie 5) and absence of nutrients for 2 hours (Supplementary Movie 6) or 24 hours (Supplementary Movie 7). n=20 cells each; 3 biological replicates each. Bar, 10 µm. Playback rate, 180x.

Supplementary Movie 8. Accumulation of ss-DsRed in the perinuclear region of control cells following its release in the acute absence of nutrients following overexpression of GFP-Sec23b. n=20 cells; 3 biological replicates. Bar, 10 µm. Playback rate, 180x.
