# Supplementary material for: Nutrient deprivation alters the rate of COPII subunit recruitment at ER subdomains to tune secretory protein transport
Source: Nat Commun. 2023 Dec 8;14:8140. doi: 10.1038/s41467-023-44002-7 (PMC10709328; doi:10.1038/s41467-023-44002-7)
Supplement: Supplementary file 13 — Source Data [file 41467_2023_44002_MOESM13_ESM.zip › Source data_NCOMMS-23-09528B/Gels and blots_labeled.pdf]

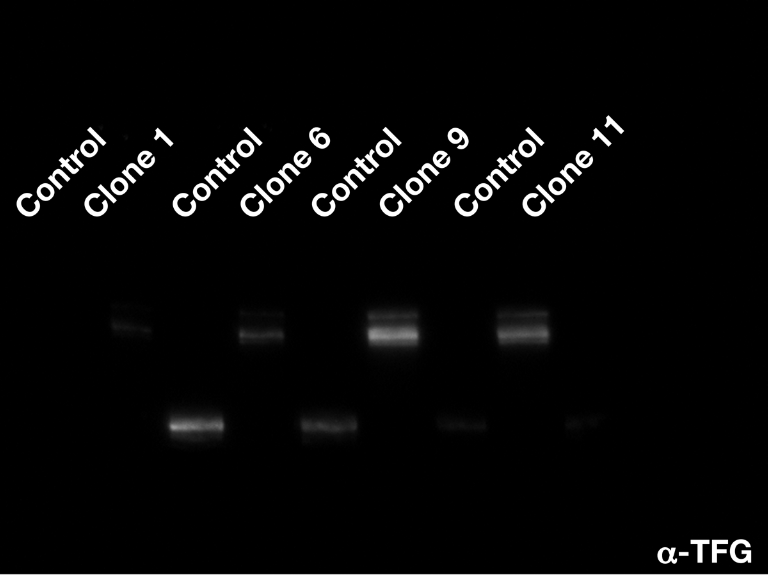
Control
Clone 1
Control
Clone 6
Control
Clone 9
Control
Clone 11
α-TFG

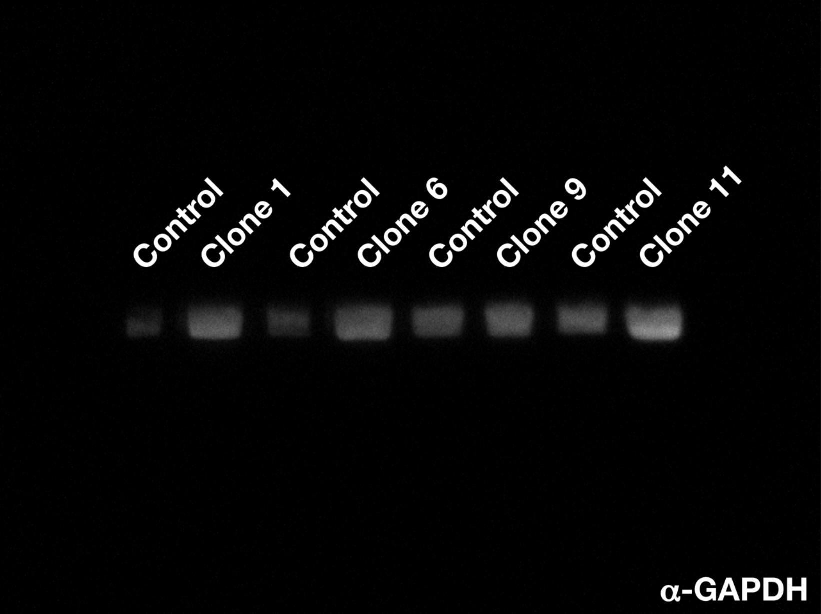
Control
Clone 1
Control
Clone 6
Control
Clone 9
Control
Clone 11
α-GAPDH

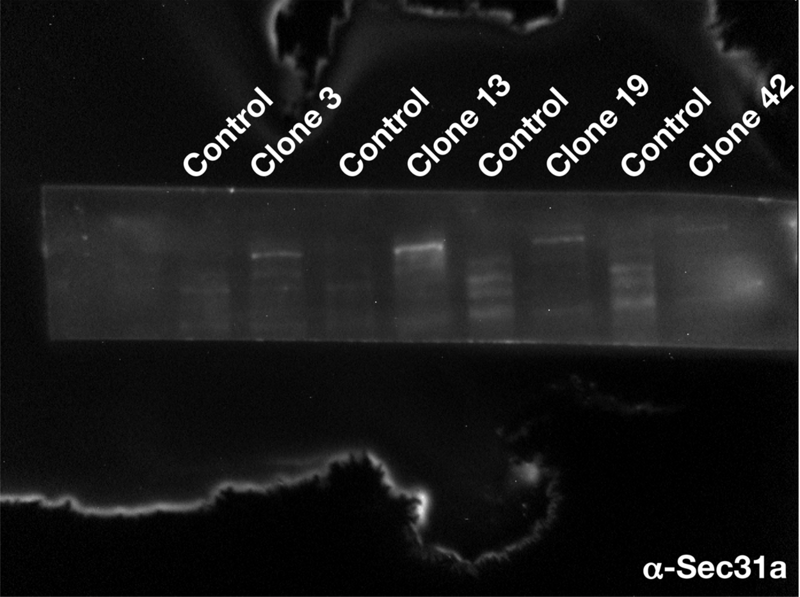
Control
Clone 3
Control
Clone 13
Control
Clone 19
Control
Clone 42
α-Sec31a

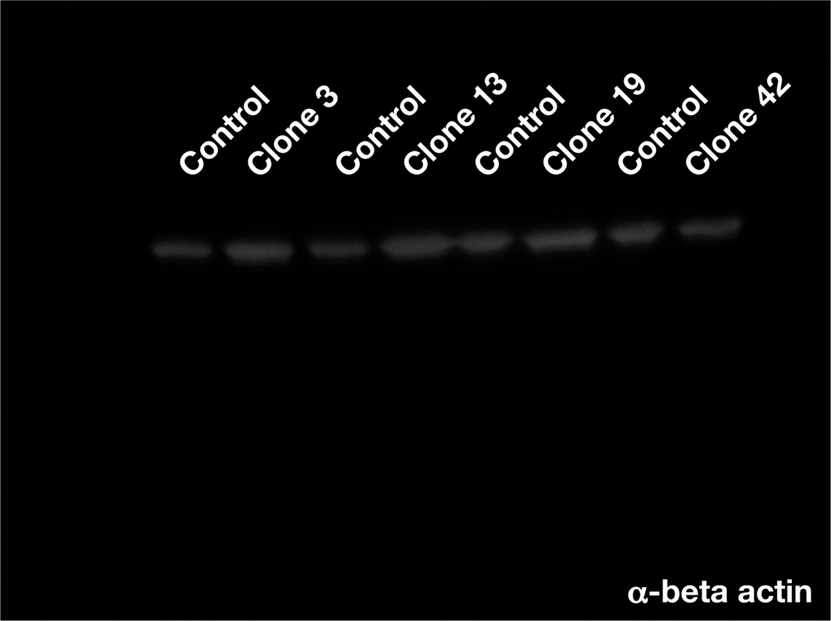
Control
Clone 3
Control
Clone 13
Control
Clone 19
Control
Clone 42
α-beta actin

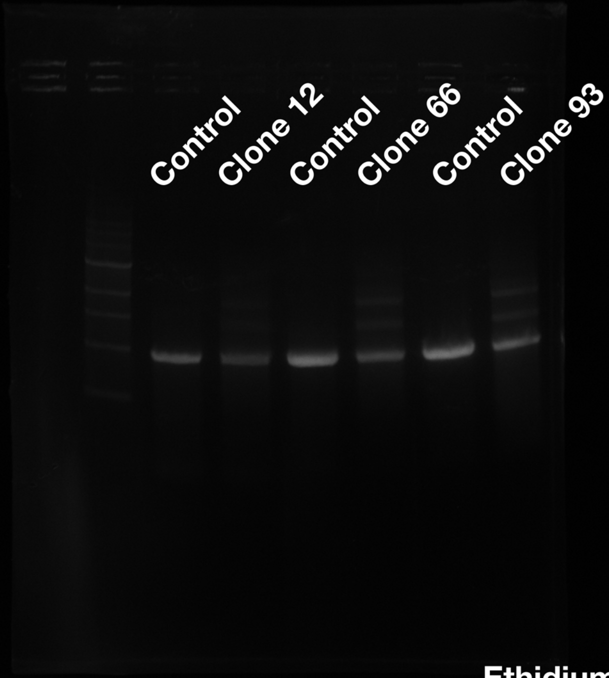

Ethidium Bromide

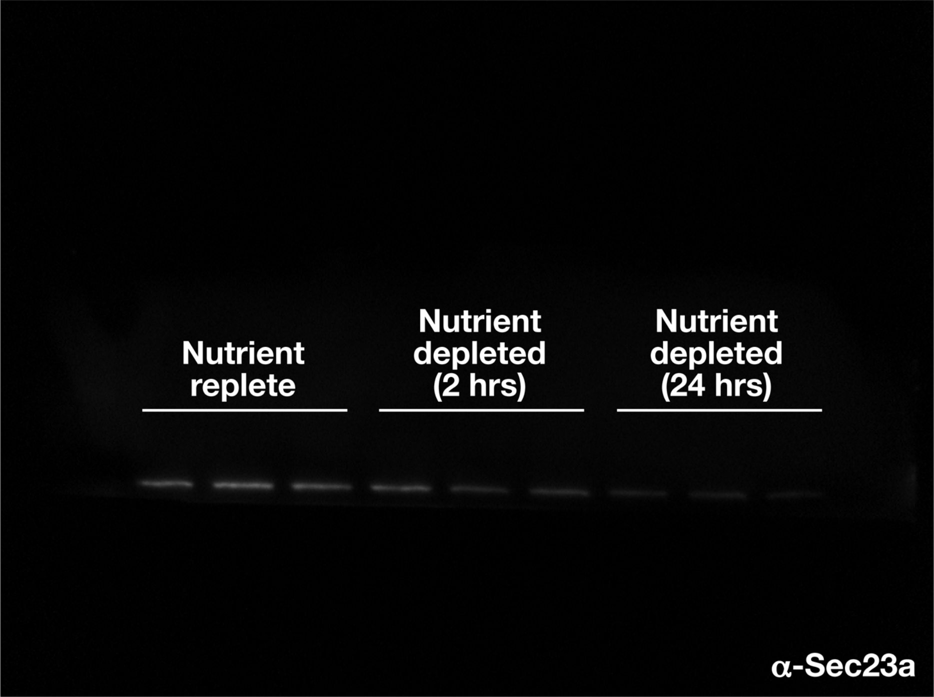
Nutrient replete
Nutrient depleted (2 hrs)
Nutrient depleted (24 hrs)
α-Sec23a

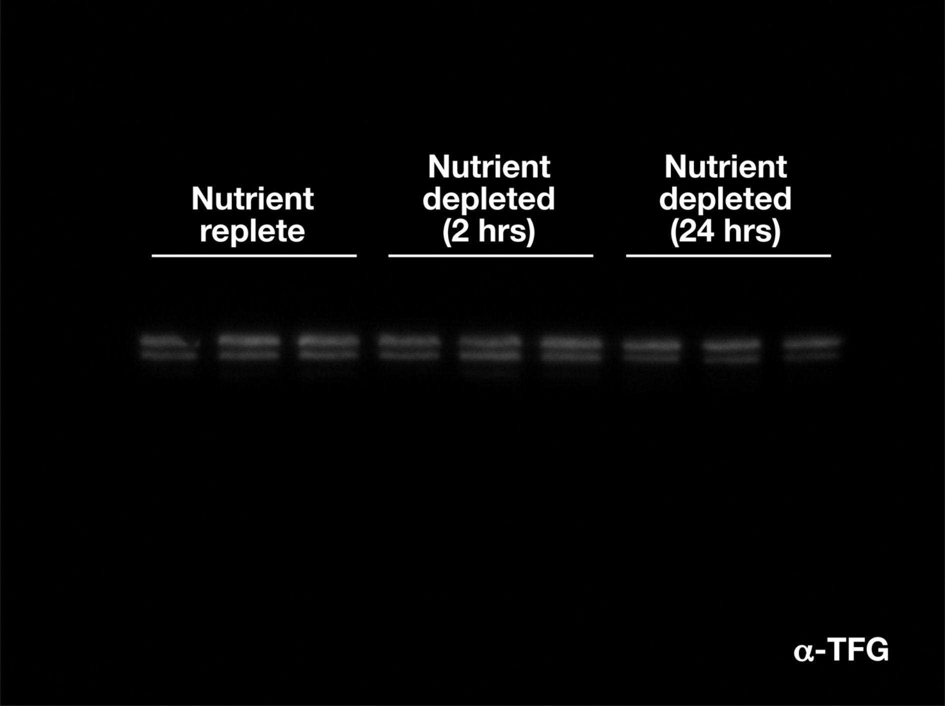

Nutrient replete
Nutrient depleted (2 hrs)
Nutrient depleted (24 hrs)
α-TFG

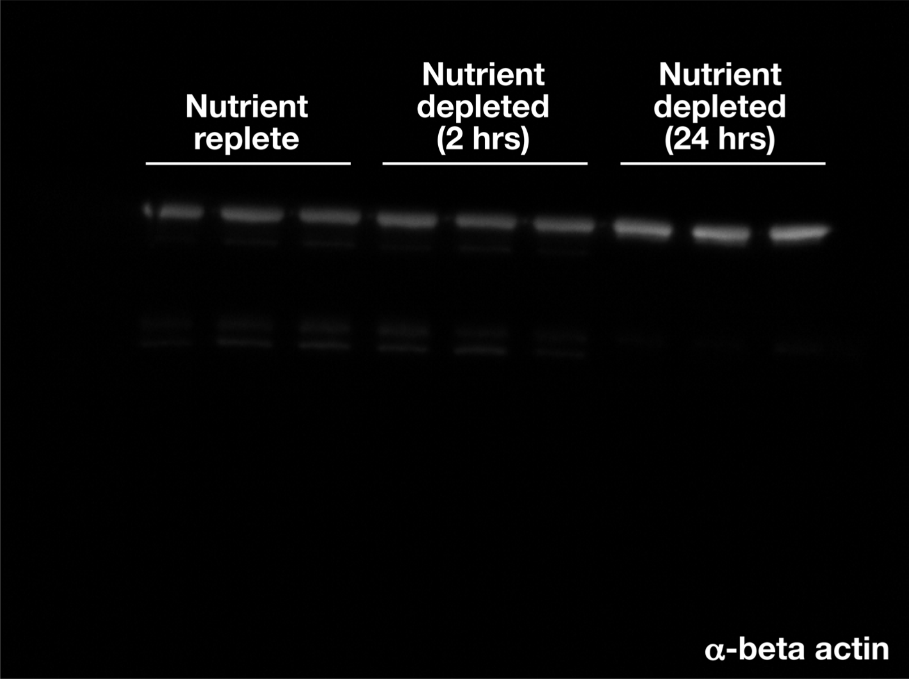
Nutrient replete
Nutrient depleted (2 hrs)
Nutrient depleted (24 hrs)
α-beta actin
