# Supplementary material for: Nutrient deprivation alters the rate of COPII subunit recruitment at ER subdomains to tune secretory protein transport
Source: Nat Commun. 2023 Dec 8;14:8140. doi: 10.1038/s41467-023-44002-7 (PMC10709328; doi:10.1038/s41467-023-44002-7)
Supplement: Supplementary file 13 — Source Data [file 41467_2023_44002_MOESM13_ESM.zip › Source data_NCOMMS-23-09528B/Figure S1C_ethidium bromide_labeled.pdf]

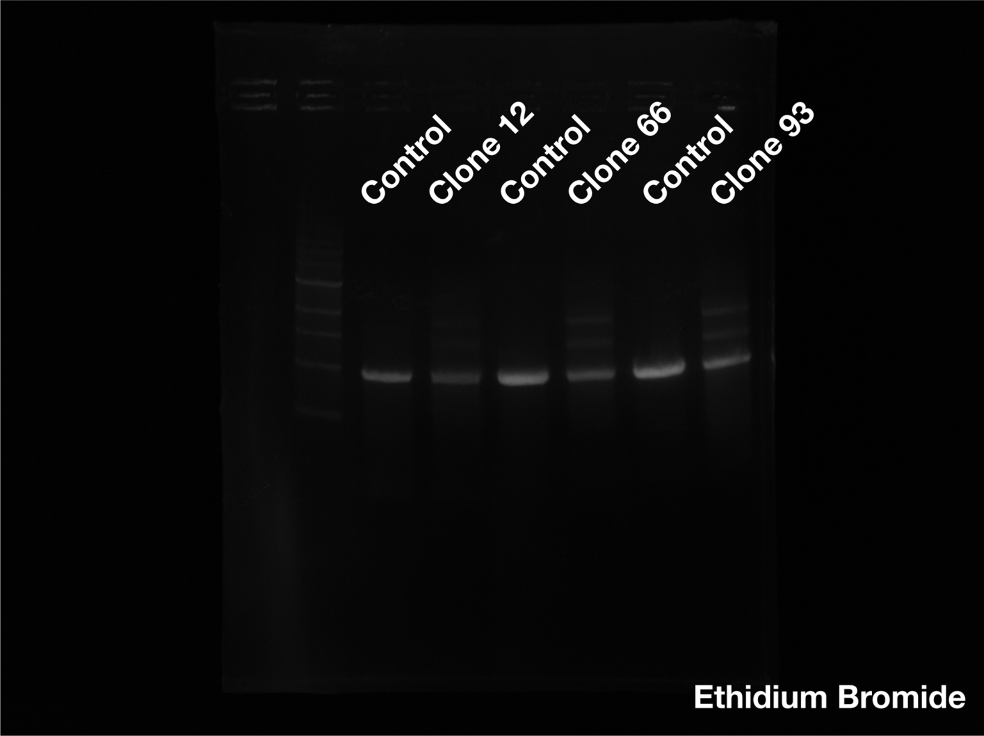
Control
Clone 12
Control
Clone 66
Control
Clone 93
Ethidium Bromide
