# Supplementary figures and images for: Nutrient deprivation alters the rate of COPII subunit recruitment at ER subdomains to tune secretory protein transport
Source: Nat Commun. 2023 Dec 8;14:8140. doi: 10.1038/s41467-023-44002-7 (PMC10709328; doi:10.1038/s41467-023-44002-7)

Control  
Clone 1  
Control  
Clone 6  
Control  
Clone 9  
Control  
Clone 11

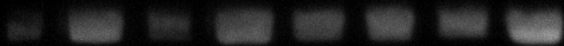

$\alpha$ -GAPDH

Supplement: Supplementary file 13 — Source Data [file 41467_2023_44002_MOESM13_ESM.zip › Source data_NCOMMS-23-09528B/Figure 1A_anti-GAPDH blot_labeled.pdf]

**Nutrient  
replete**

---

**Nutrient  
depleted  
(2 hrs)**

---

**Nutrient  
depleted  
(24 hrs)**

---

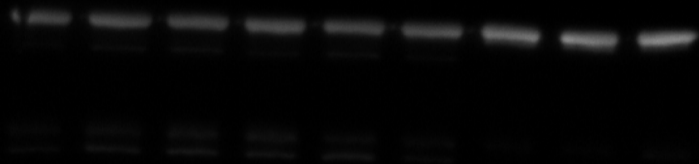

**$\alpha$ -beta actin**

Supplement: Supplementary file 13 — Source Data [file 41467_2023_44002_MOESM13_ESM.zip › Source data_NCOMMS-23-09528B/Figure S8C_anti-actin blot_labeled.pdf]

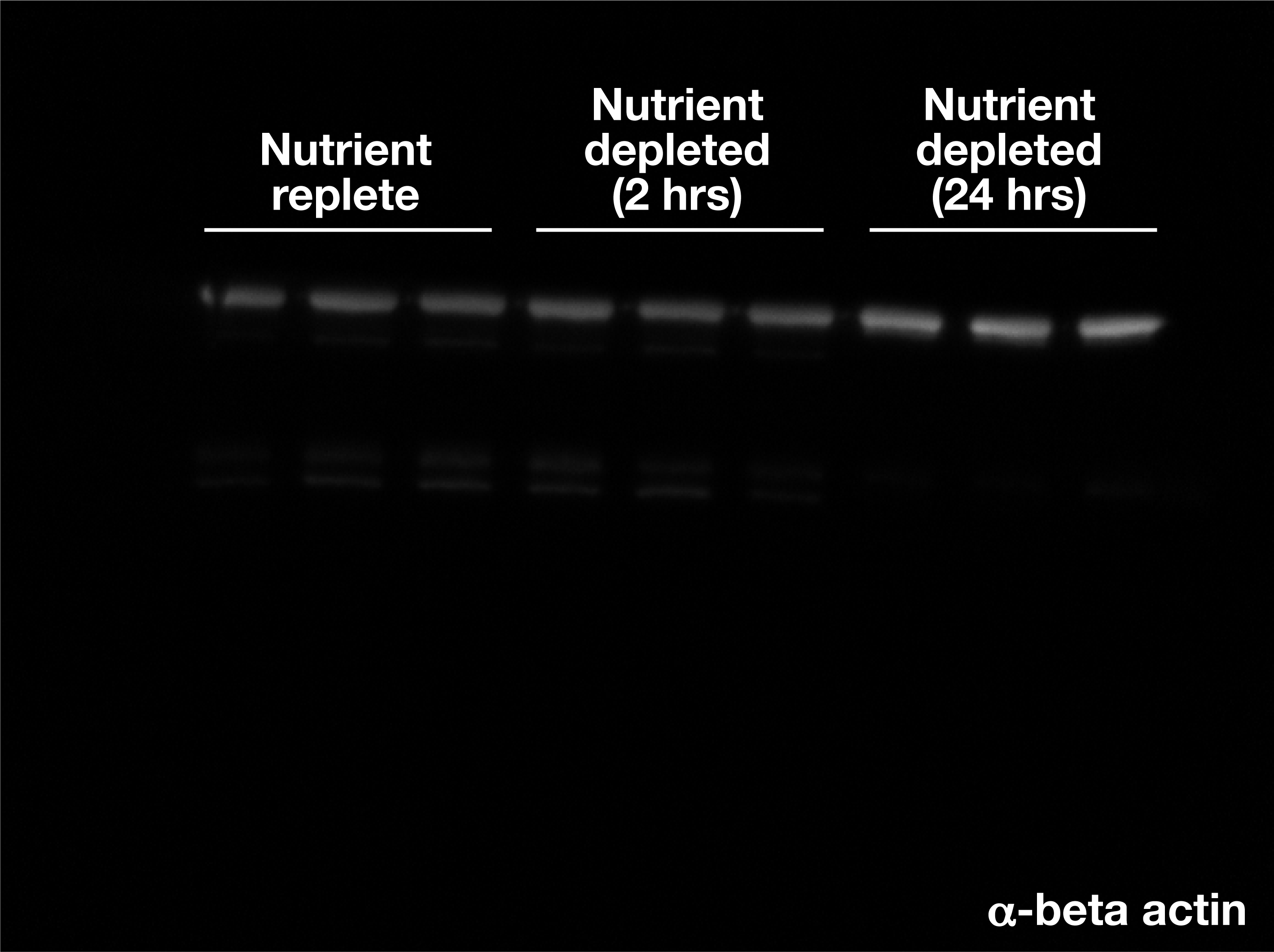

Supplement: Supplementary file 13 — Source Data [file 41467_2023_44002_MOESM13_ESM.zip › Source data_NCOMMS-23-09528B/Figure S8C_anti-actin blot_labeled.tif]

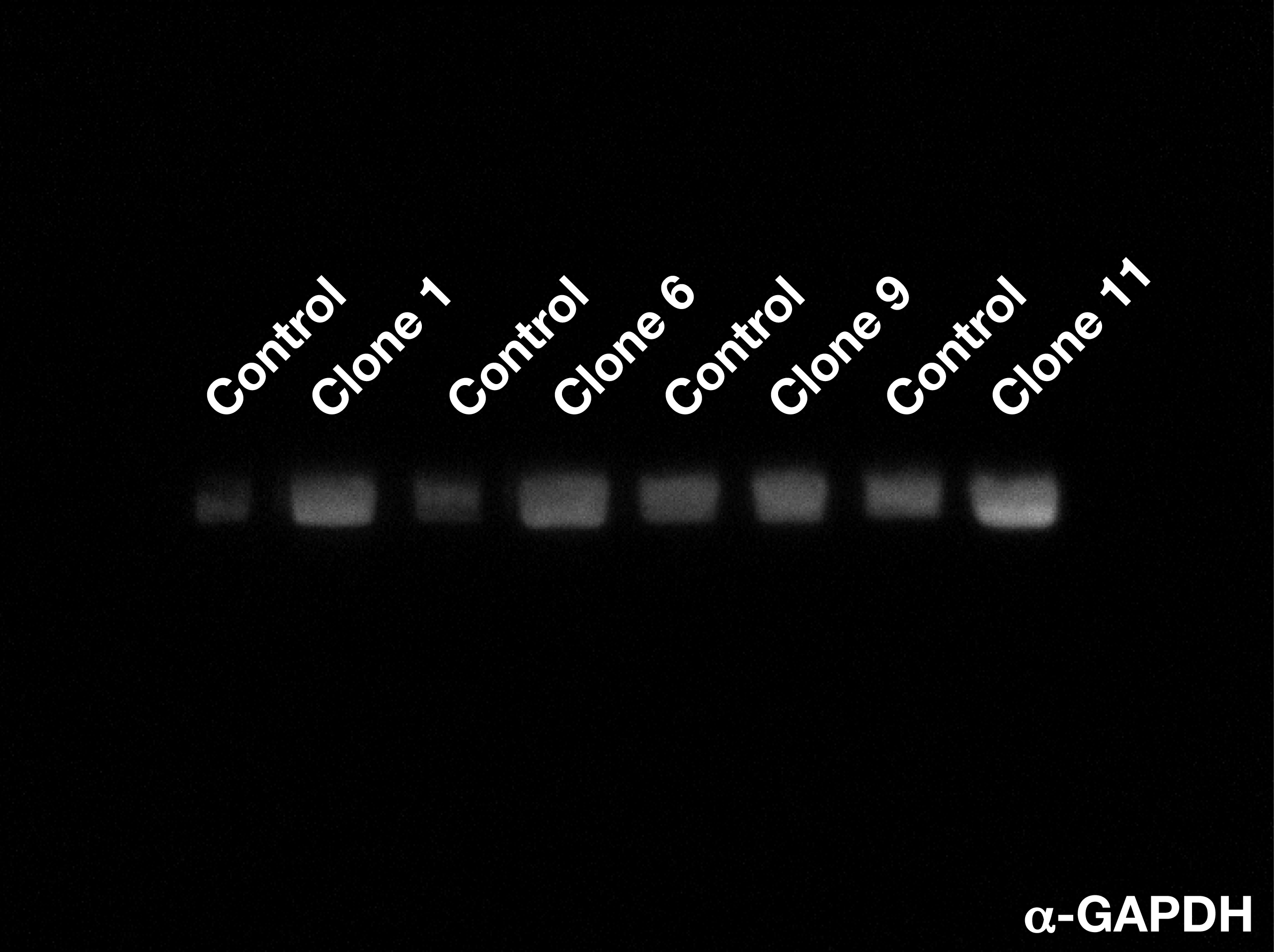

Supplement: Supplementary file 13 — Source Data [file 41467_2023_44002_MOESM13_ESM.zip › Source data_NCOMMS-23-09528B/Figure 1A_anti-GAPDH blot_labeled.tif]

**Nutrient  
replete**

---

**Nutrient  
depleted  
(2 hrs)**

---

**Nutrient  
depleted  
(24 hrs)**

---

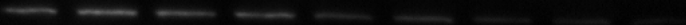

**$\alpha$ -Sec23a**

Supplement: Supplementary file 13 — Source Data [file 41467_2023_44002_MOESM13_ESM.zip › Source data_NCOMMS-23-09528B/Figure S8C_anti-Sec23 blot_labeled.pdf]

**Nutrient  
replete**

---

**Nutrient  
depleted  
(2 hrs)**

---

**Nutrient  
depleted  
(24 hrs)**

---

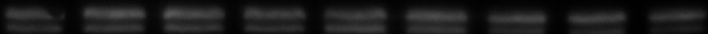

**$\alpha$ -TFG**

Supplement: Supplementary file 13 — Source Data [file 41467_2023_44002_MOESM13_ESM.zip › Source data_NCOMMS-23-09528B/Figure S8C_anti-TFG blot_labeled.pdf]

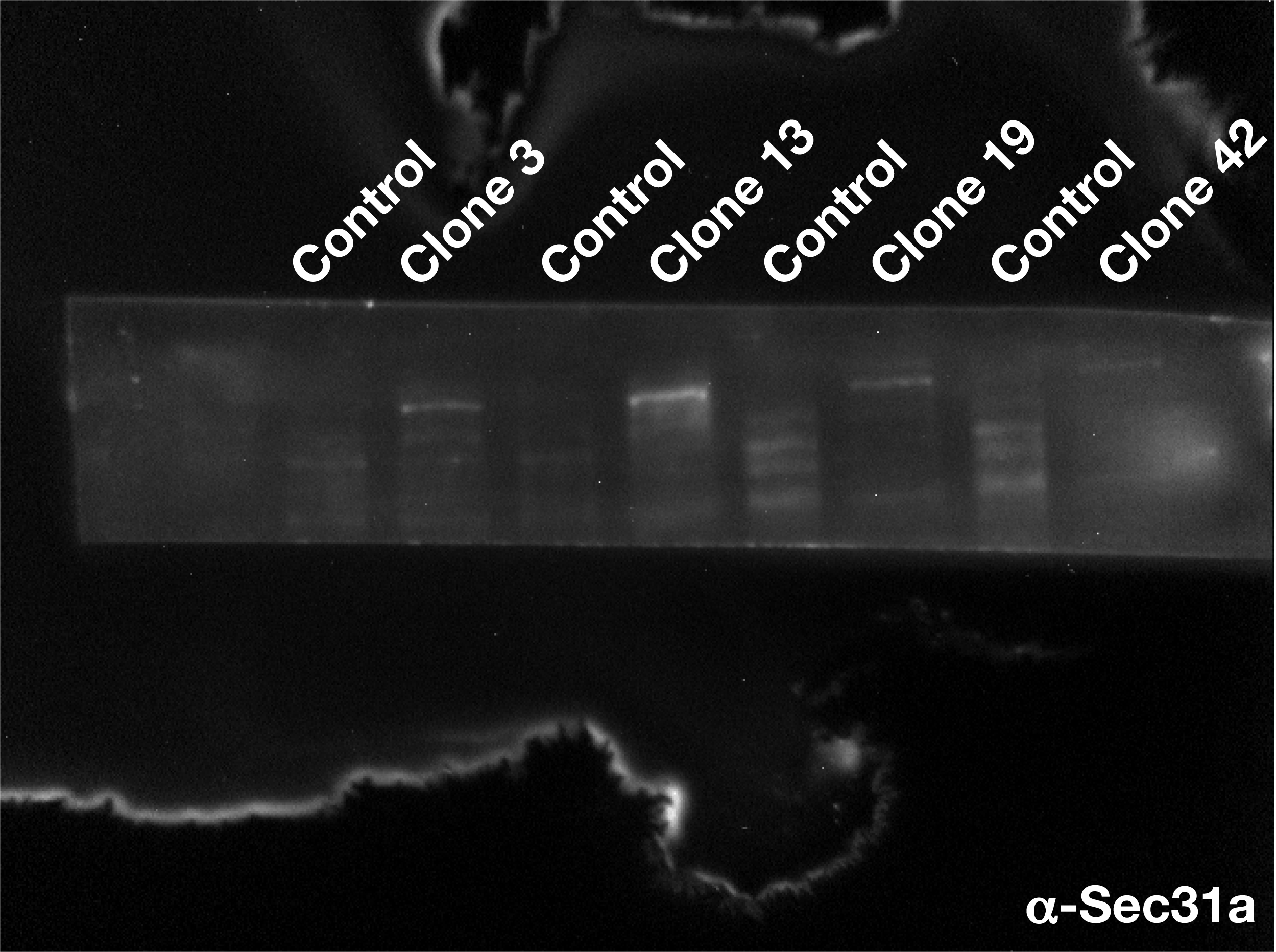

Supplement: Supplementary file 13 — Source Data [file 41467_2023_44002_MOESM13_ESM.zip › Source data_NCOMMS-23-09528B/Figure S1B_anti-Sec31a blot_labeled.tif]

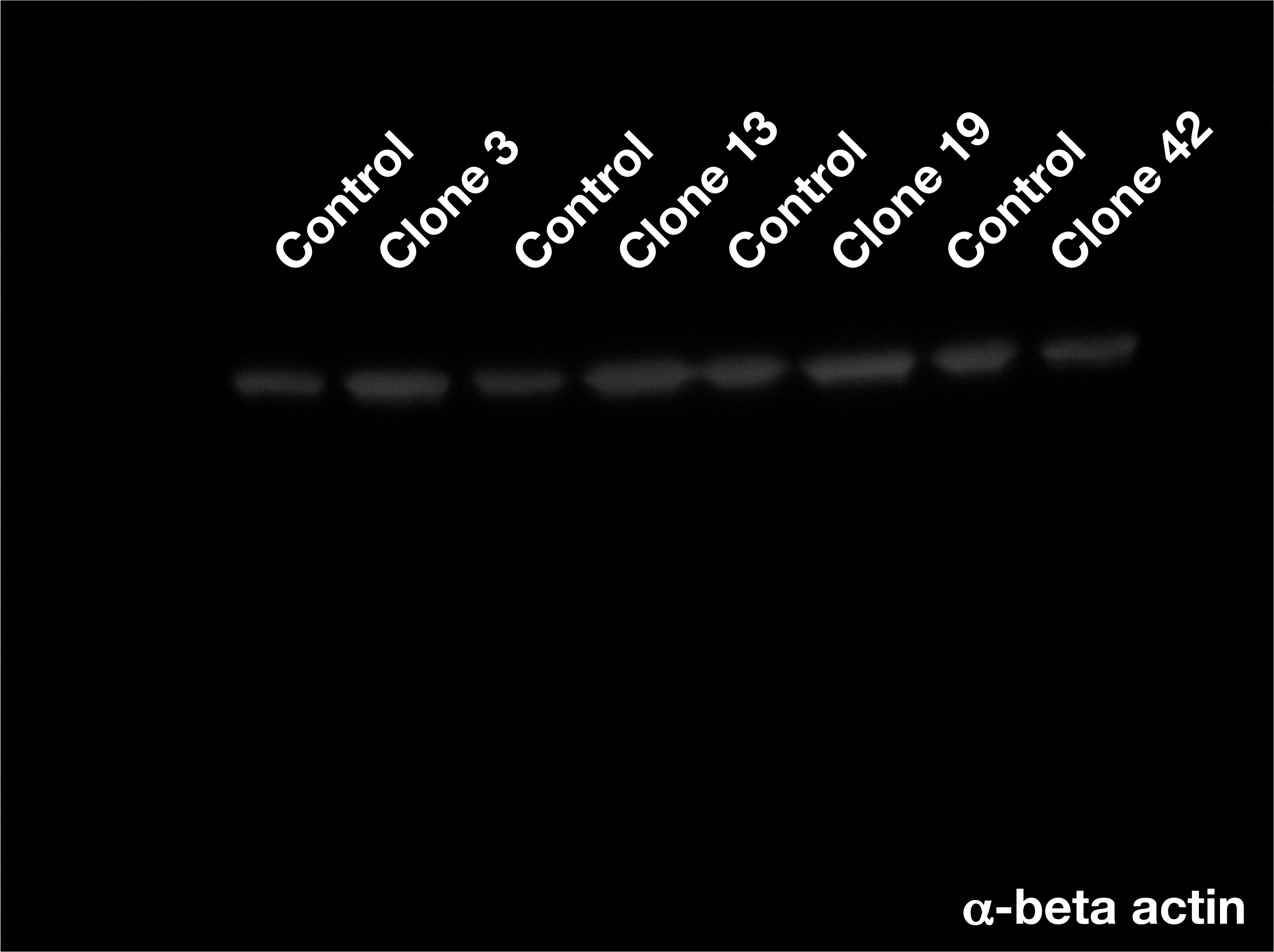

Supplement: Supplementary file 13 — Source Data [file 41467_2023_44002_MOESM13_ESM.zip › Source data_NCOMMS-23-09528B/Figure S1B_anti-actin blot_labeled.tif]

Control  
Clone 1

Control  
Clone 6

Control  
Clone 9

Control  
Clone 11

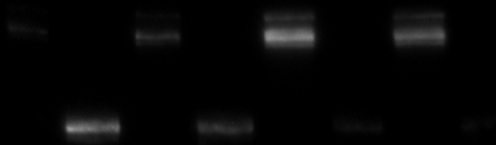

$\alpha$ -TFG

Supplement: Supplementary file 13 — Source Data [file 41467_2023_44002_MOESM13_ESM.zip › Source data_NCOMMS-23-09528B/Figure 1A_anti-TFG blot_labeled.pdf]

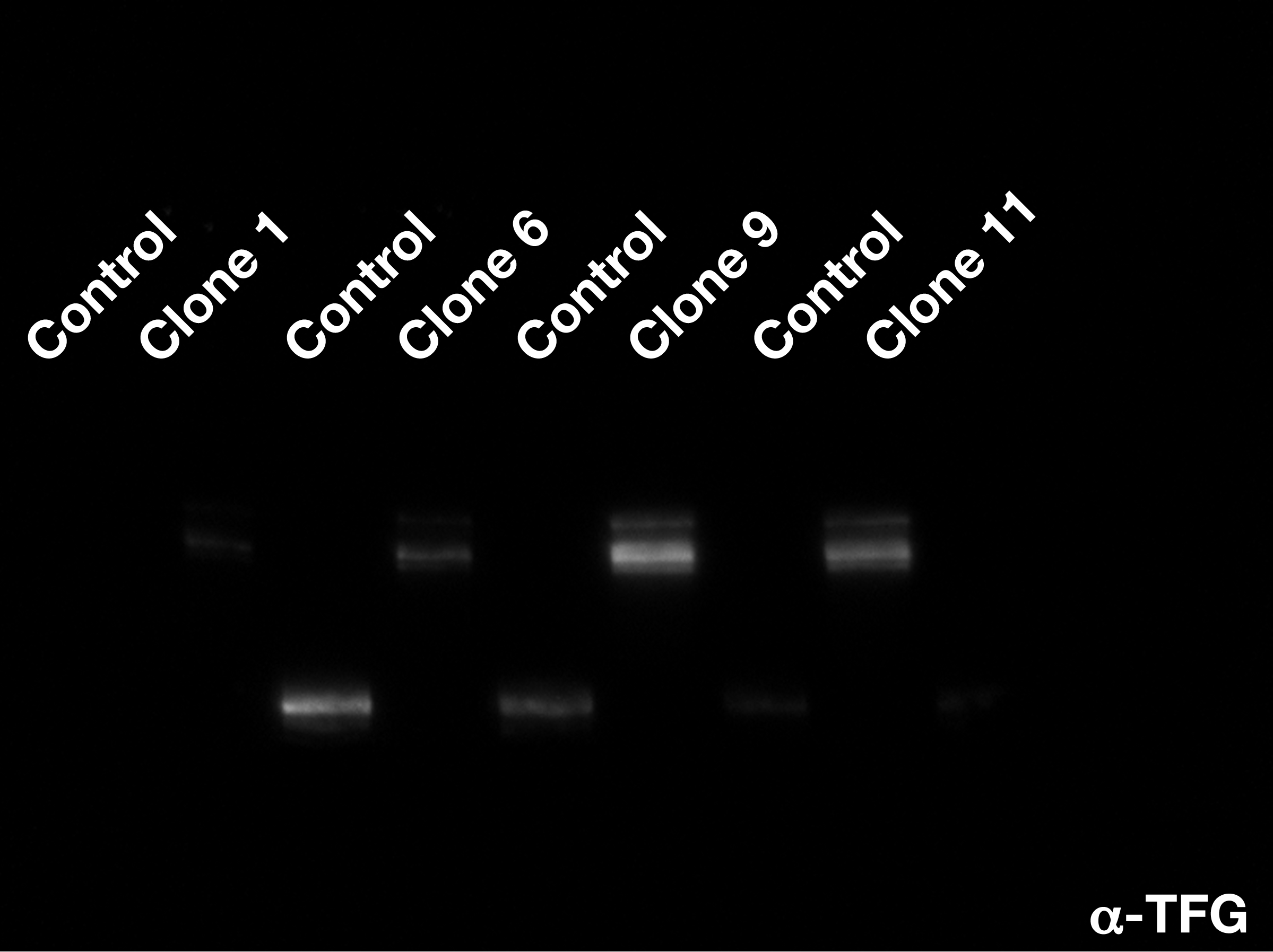

Supplement: Supplementary file 13 — Source Data [file 41467_2023_44002_MOESM13_ESM.zip › Source data_NCOMMS-23-09528B/Figure 1A_anti-TFG blot_labeled.tif]

Control  
Clone 3

Control  
Clone 13

Control  
Clone 19

Control  
Clone 42

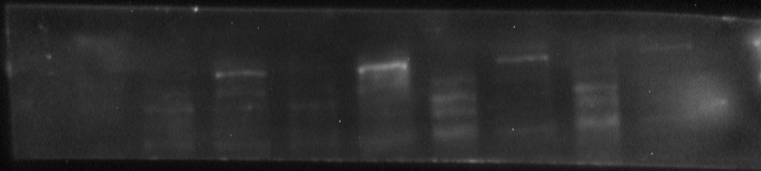

$\alpha$ -Sec31a

Supplement: Supplementary file 13 — Source Data [file 41467_2023_44002_MOESM13_ESM.zip › Source data_NCOMMS-23-09528B/Figure S1B_anti-Sec31a blot_labeled.pdf]

Control Clone 3 Control Clone 13 Control Clone 19 Control Clone 42

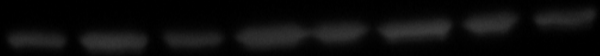

$\alpha$ -beta actin

Supplement: Supplementary file 13 — Source Data [file 41467_2023_44002_MOESM13_ESM.zip › Source data_NCOMMS-23-09528B/Figure S1B_anti-actin blot_labeled.pdf]

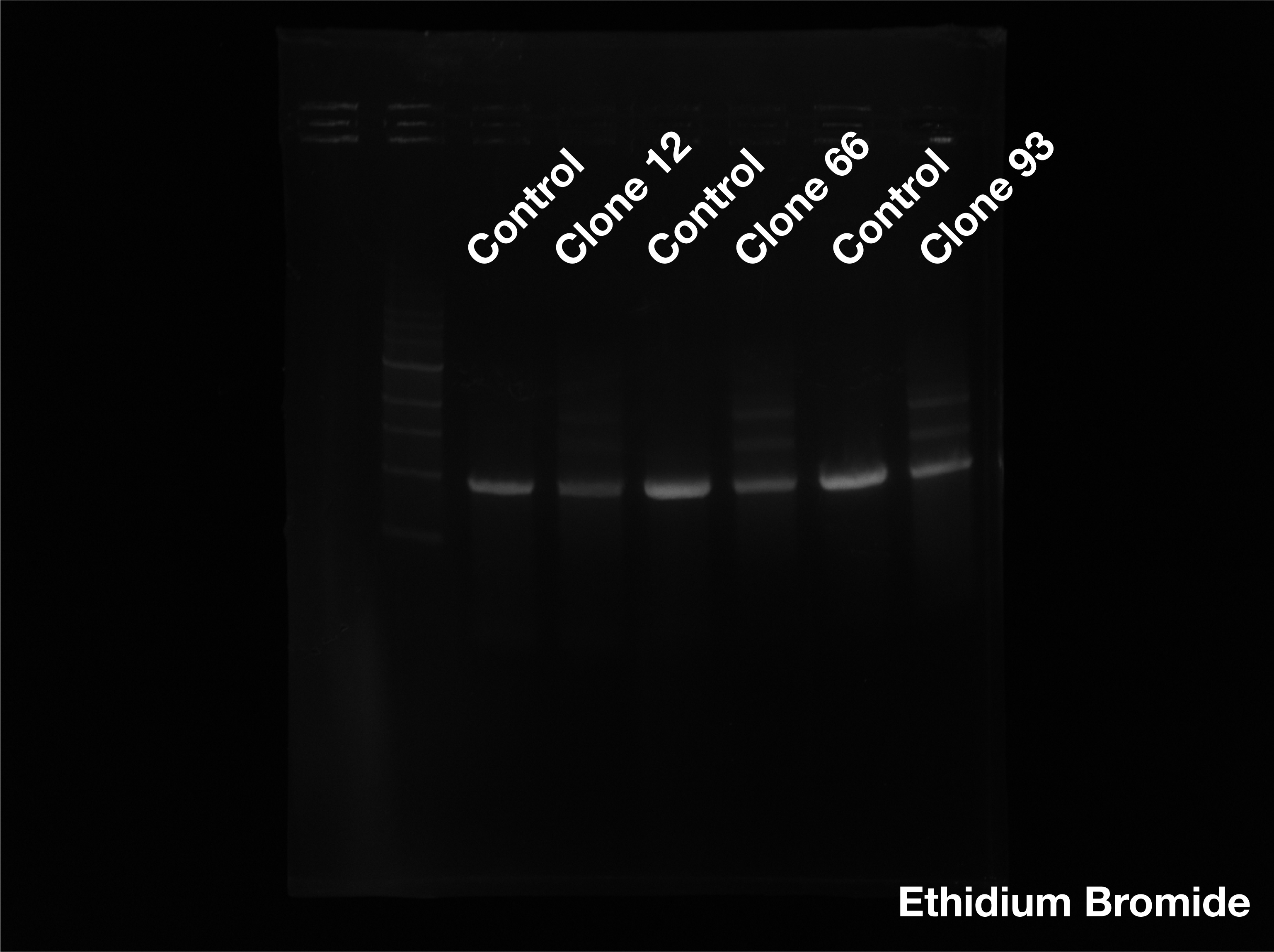

Supplement: Supplementary file 13 — Source Data [file 41467_2023_44002_MOESM13_ESM.zip › Source data_NCOMMS-23-09528B/Figure S1C_ethidium bromide_labeled.tif]

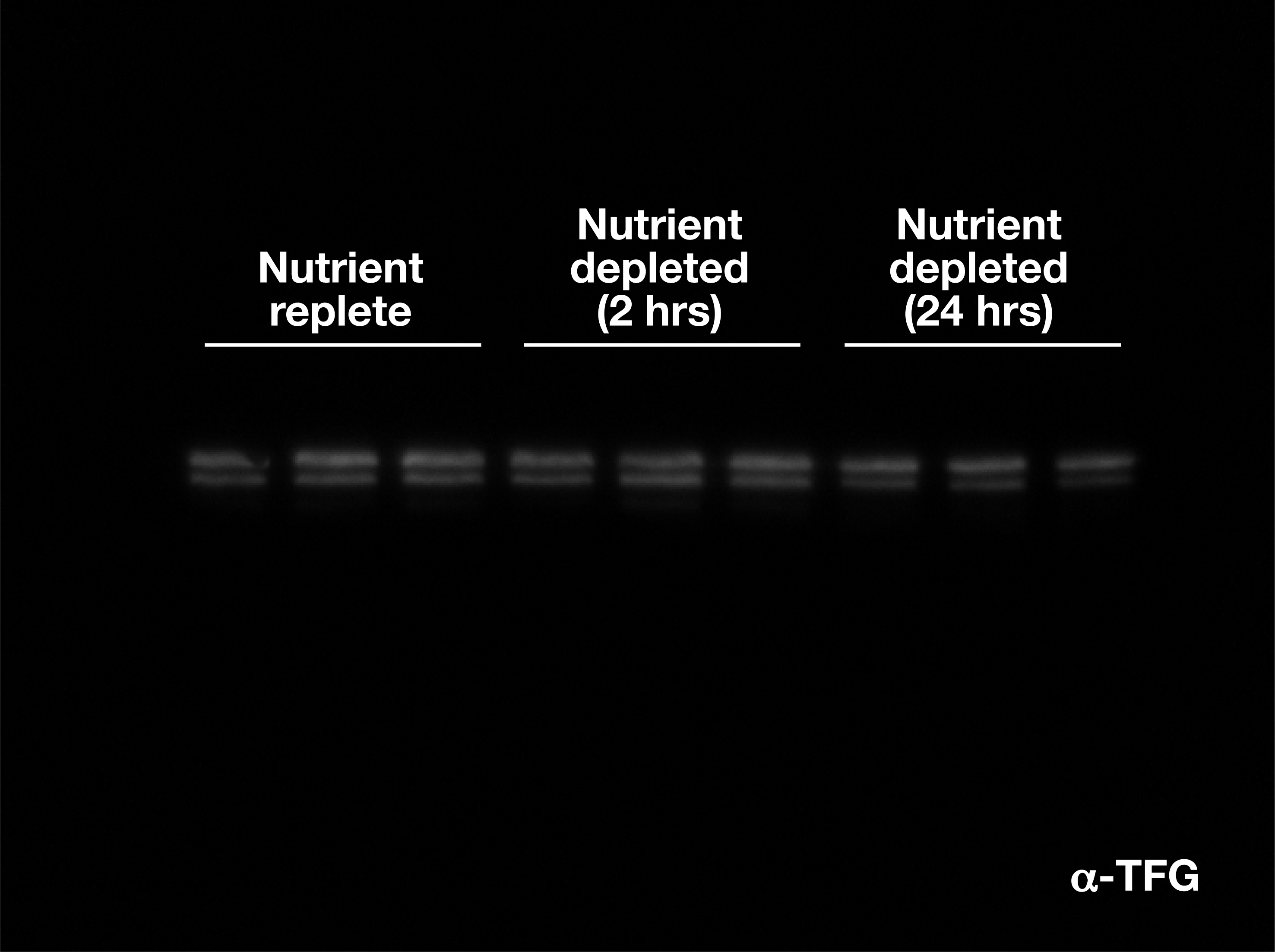

Supplement: Supplementary file 13 — Source Data [file 41467_2023_44002_MOESM13_ESM.zip › Source data_NCOMMS-23-09528B/Figure S8C_anti-TFG blot_labeled.tif]

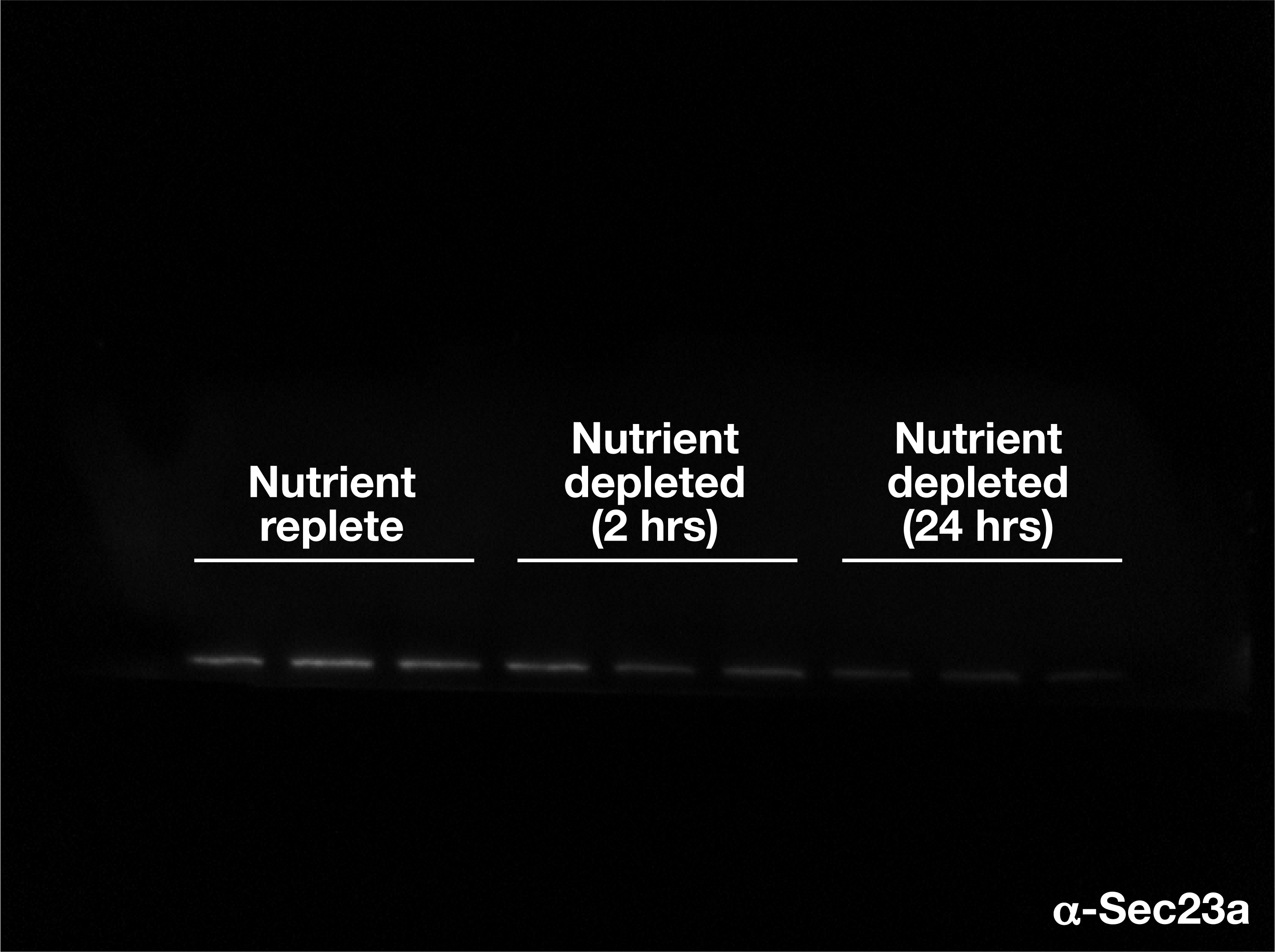

Supplement: Supplementary file 13 — Source Data [file 41467_2023_44002_MOESM13_ESM.zip › Source data_NCOMMS-23-09528B/Figure S8C_anti-Sec23 blot_labeled.tif]
